# Reactivity of Tetrel-Functionalized Heptaphosphane Clusters toward Azides

**DOI:** 10.1021/acs.inorgchem.4c02264

**Published:** 2024-07-16

**Authors:** William D. Jobbins, Rory T. Cullen, Thomas Stott, Bono van IJzendoorn, Benjamin L. L. Réant, Timothy C. Johnstone, Meera Mehta

**Affiliations:** † Department of Chemistry, University of Manchester, Oxford Road, Manchester M13 9PL, U.K.; ‡ Department of Chemistry, University of Oxford, 12 Mansfield Road, Oxford OX1 3TA, U.K.; § Department of Chemistry and Biochemistry, University of California, Santa Cruz, 1156 High Street, Santa Cruz, California 95064, United States

## Abstract

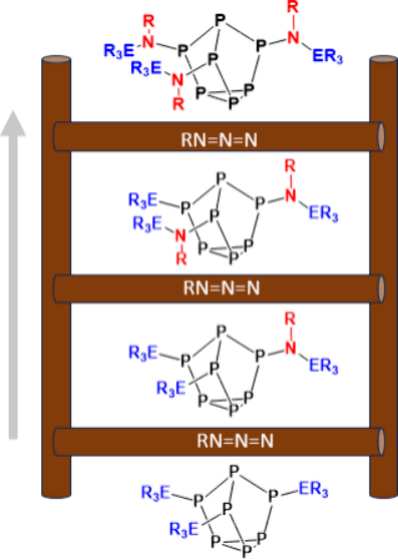

In this work, the reactivity of tetrel-functionalized phosphorus clusters toward organoazides is probed. Clusters (Me_3_Si)_3_P_7_ (**1**) and (Me_3_Ge)_3_P_7_ (**2**) were reacted with benzyl azide, phenyl azide, and 4-bromophenyl azide, and it was found that the [RN] (R = benzyl, phenyl, and 4-bromophenyl) unit from the azide inserted into the phosphorus–tetrel bonds on the cluster, accompanied by N_2_ elimination. Through control of the azide stoichiometry, the mono-, bis-, and tris-inserted products could be observed, consistent with these insertions proceeding in a stepwise manner. The bonding between the amine moieties and clusters was further investigated by computational chemistry, and the findings were consistent with the phosphorus cluster having undergone a formal oxidation. These insertion reactions are a convenient means of accessing Zintl clusters functionalized with *exo*-nitrogen-bonded moieties, which, to the best of our knowledge, were previously unknown.

## Abstract 2

Delivered from organoazides, [RN] (R = benzyl, phenyl, and 4-bromophenyl) units are inserted between the P−E (E = Si, Ge) bonds of (Me_3_E)_3_P_7_ clusters. These insertions afford nitrogen-bonded moieties at the cluster, which results in a reversal of the phosphorus−element bond dipole. Further, through control of the stoichiometry, the mono- and bis-, and tris-inserted products could be identified.

## Introduction

There has been a reignited interest in studying the chemistry of Zintl clusters, given their interesting bonding and aesthetically pleasing architectures.^1^ Recent developments in the field have been focused on probing their coordination chemistry with transition metals to allow access to new physical properties,^2^ applying them as catalysts to mediate organic transformations,^3,4^ and using them as precursors in the bottom-up solution-state synthesis of nanostructures.^5^ Although the applications of these clusters can often be tuned by functionalizing them with organic units, the number of functionalization options is unfortunately limited.

**Figure 1:**
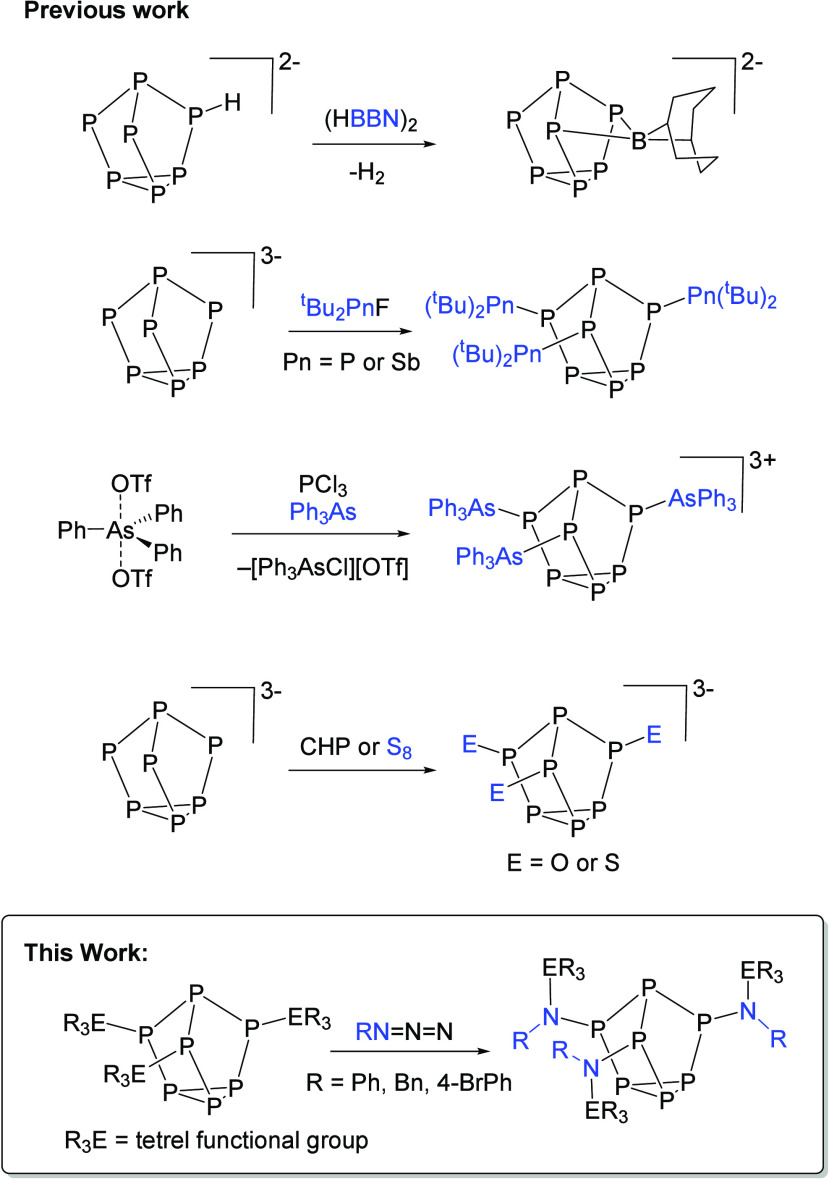
Select examples of [P_7_] functionalization and this work (CHP = cumene hydroperoxide).

One cluster that has been widely studied is the heptaphosphanortricyclanide trianion, [P_7_]^3–^, in part due to its ease of synthesis and the presence of the ^31^P NMR-active handle, which enables *in situ* studies by NMR spectroscopy.^6^ Historically, the anionic nature of this cluster has been exploited in reactions with Brønsted acids to give [HP_7_]^2–^, [H_2_P_7_]^−^, and [H_3_P_7_] and in salt elimination reactions that functionalize the cluster with organotetrel groups or transition metals.^6−8^ In 2022, we reported on the first fully characterized boron-functionalized Group 15 cluster by dehydrocoupling 9-borabicyclo[3.3.1]nonane with [HP_7_]^2–^ (Figure 1).^4^ Generally, given the electron-rich nature of these phosphorus clusters, functionalization with moieties where the cluster-bonded element is from Groups 15 or 16 is difficult. In one of the few known cases, Fritz and co-workers prepared phosphorus- and antimony-functionalized [P_7_] clusters by reacting Li_3_P_7_ with either ^t^Bu_2_PF or ^t^Bu_2_SbCl, correspondingly.^9^ In another case, Weigand and co-workers formed the arsenic-functionalized system [(AsPh_3_)_3_P_7_][OTf]_3_ by reductive coupling of PCl_3_ with AsPh_3_ and Ph_3_As(OTf)_2_.^10^ Baudler et al. accessed chalcogen-functionalized clusters by reacting Li_3_P_7_ with cumene hydroperoxide^11^ and elemental sulfur (S_8_),^12^ although these oxidized Zintl cluster products were only characterized by NMR spectroscopy.

Insertion reactions are an alternative methodology for functionalization, and in 2022 and 2023, we reported that isocyanates can be inserted between the tetrel–pnictogen bonds of (R_3_E)_3_Pn_7_ (E = Si, Ge, Sn; Pn = P, As) to generate [Pn_7_] clusters that feature carbonyl moieties.^13,14^ Goicoechea and co-workers reported similar isocyanate and carbodiimide insertions into the H–P bonds of protonated clusters.^8,15^ Here we report that organoazides (RN_3_) react with (R_3_E)_3_P_7_ (E = Si, Ge) to eliminate N_2_ and insert [RN] units between the E–P bonds of the cluster (Figure 1). Although azides have been extensively used to transfer [RN] units in noncluster chemistry (e.g., Staudinger reactions^16^), this is the first exploration of such reactivity within Zintl chemistry. Furthermore, to the best of our knowledge, this convenient method allows for the preparation of a unique class of Zintl clusters with *exo*-nitrogen-bonded functional groups. Installing moieties where P–N bonds are generated at these clusters is interesting because nitrogen has a higher Pauling electronegativity than phosphorus,^17^ which is expected to reverse the dipole of this bond compared to the P–E bonds where E is a less electronegative element, including Si, Ge, Sn, Sb, and As.

## Results and Discussion

**Scheme 1:**
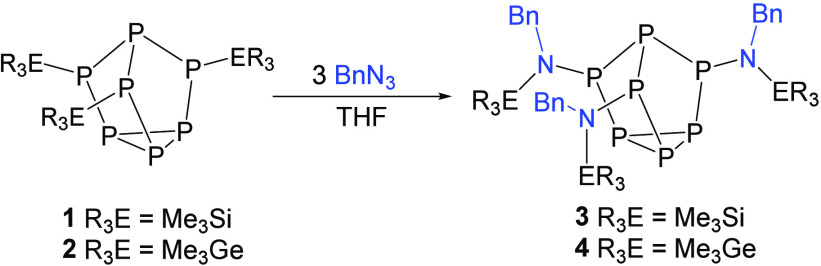
Reaction of Benzyl Azide with Clusters **1** and **2**

First, [Na(DME)_*x*_]_3_[P_7_] (DME = dimethoxyethane) was prepared using the method reported by Grützmacher and co-workers.^18^ Next, following literature protocols, the tetrel-functionalized clusters (Me_3_Si)_3_P_7_ (**1**) and (Me_3_Ge)_3_P_7_ (**2**) shown in Scheme 1 were prepared via salt elimination of [Na(DME)_*x*_]_3_[P_7_] with the corresponding tetrel chloride, Me_3_ECl (E = Si, Ge).^13,14^ These tetrel-functionalized clusters were then allowed to react with organoazides, including benzyl azide (BnN_3_), phenyl azide (PhN_3_), and 4-bromophenyl azide (4-BrPhN_3_).

**Figure 2:**
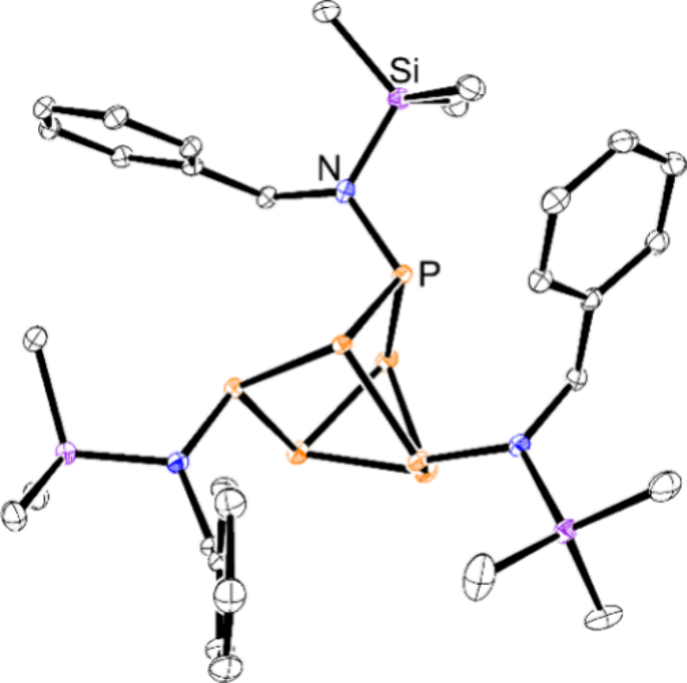
Molecular structure of **3**. Anisotropic displacement ellipsoids pictured at 50% probability. Hydrogen atoms have been omitted for clarity. Color code: phosphorus, orange; silicon, purple; nitrogen, blue; carbon, black.

When a tetrahydrofuran (THF) solution of **1** was allowed to react with a solution of 3 equiv of BnN_3_, an immediate color change from pale yellow to deeper yellow was observed along with gas evolution, which was presumed to be N_2_ (Scheme 1). Investigation of the reaction mixture by ^29^Si and ^31^P NMR spectroscopy revealed complete consumption of the starting material. Further, a new resonance was observed in the ^29^Si NMR spectrum at 14.9 ppm with a ^2^*J*_SiP_ coupling constant of 27.9 Hz, significantly smaller than the ^1^*J*_SiP_ coupling constant of 42.0 Hz observed for **1**. Additionally, three new signals were observed in the ^31^P NMR spectrum, consistent with the basal, apical, and bridging phosphorus atoms of a new cluster product. Single crystals were grown, and subsequent X-ray diffraction (XRD) studies further confirmed the solid-state structure of the product to be (Me_3_Si[BnN])_3_P_7_ (**3**, CCDC 2351073; Figure 2), where BnN_3_ had lost N_2_ and [BnN] units were inserted between all three of the Si–P bonds of the cluster. Analysis of the bond metrics revealed an average P–N bond length of 1.693(13) Å, an average N–Si bond length of 1.765(14) Å, and an average P–N–Si bond angle of 116.0(7)°.

**Figure 3:**
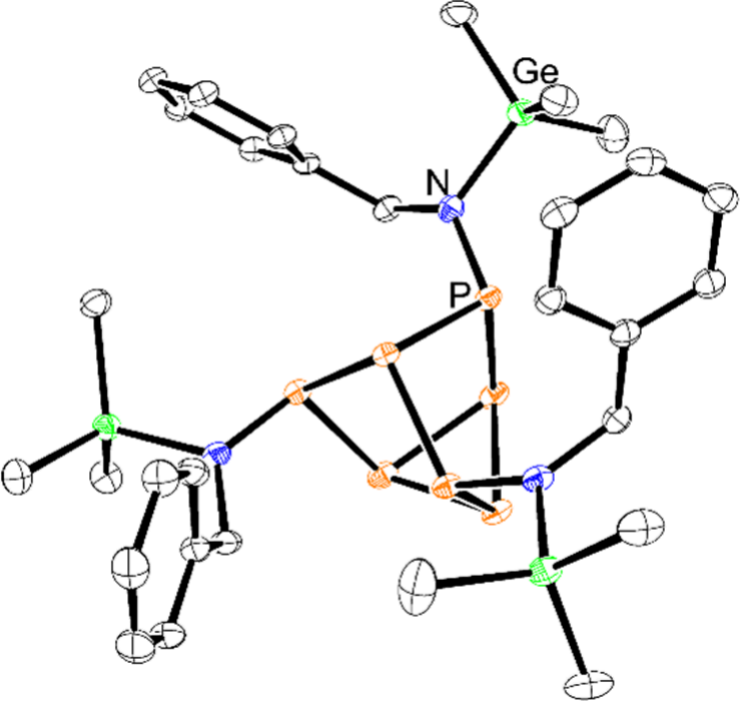
Molecular structure of **4**. Anisotropic displacement ellipsoids pictured at 50% probability. Hydrogen atoms have been omitted for clarity. Color code: phosphorus, orange; germanium, green; nitrogen, blue; carbon, black.

In a similar fashion, treatment of **2** with BnN_3_ also resulted in a color change from pale yellow to slightly darker yellow and NMR spectra consistent with the formation of a new cluster product (Scheme 1). This product was further structurally authenticated to be **4** (CCDC 2351074) using XRD (Figure 3). Analysis of the bond metrics revealed an average P–N bond length of 1.685(3) Å, which is in good agreement with standard P–N single bonds,^19^ N–Ge bond lengths of 1.878(3) Å, and P–N–Ge bond angles of 115.3(14)°. The intracluster P–P bond metrics for compounds **3** and **4** were consistent with the corresponding metrics for the starting materials **1** and **2**, showing minimal perturbation of the cluster core by introduction of the amine moieties.

**Scheme 2:**
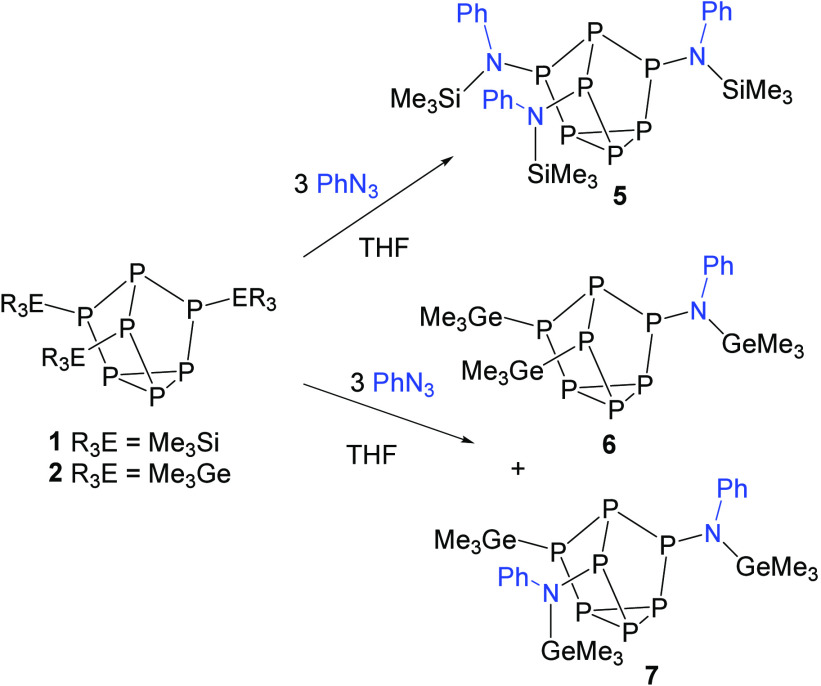
Reactions of Phenyl Azide with Clusters **1** and **2**

**Figure 4:**
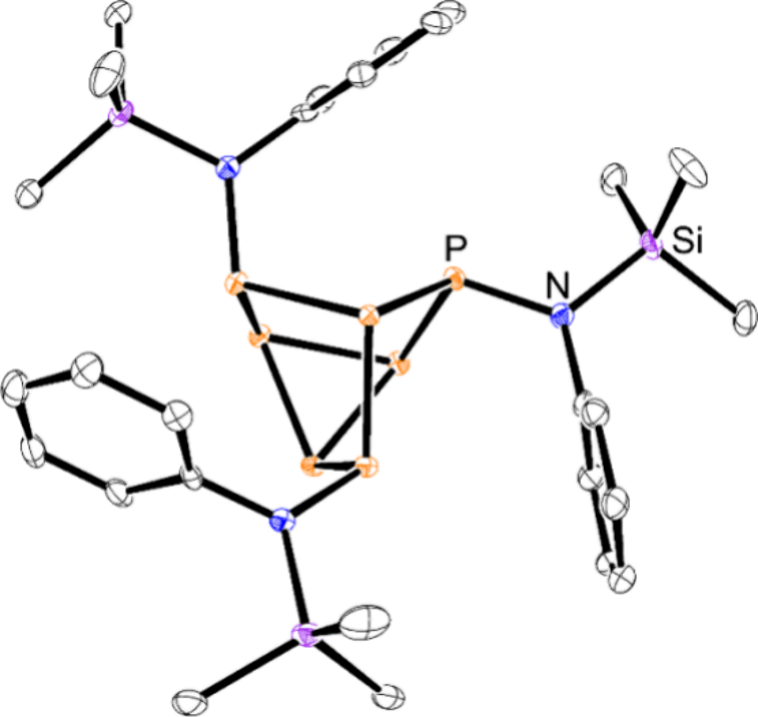
Molecular structure of **5**. Anisotropic displacement ellipsoids pictured at 50% probability. Hydrogen atoms have been omitted for clarity. Color code: phosphorus, orange; silicon, purple; nitrogen, blue; carbon, black.

Next, **1** was allowed to react with 3 equiv of PhN_3_, and akin to its reactivity with BnN_3_, a color change and gas evolution were observed (Scheme 2). As expected, analysis of the ^31^P NMR spectrum revealed the three characteristic resonances of a symmetric cluster product, and the ^29^Si NMR spectrum showed a single resonance at 12.6 ppm with a ^2^*J*_SiP_ coupling constant of 30.1 Hz. XRD studies of the isolated crystals further confirmed the structure of this new product to be **5** (CCDC 2351075; Figure 4), with tris-insertion of the [PhN] units between the three P–Si bonds of **1**. Analysis of the bond metrics from this XRD data showed an average P–N bond length of 1.699(4) Å, a N–Si bond length of 1.770(5) Å, and a P–N–Si bond angle of 118.4(7)°, consistent with the bond metrics observed for compounds **3** and **4**.

**Figure 5:**
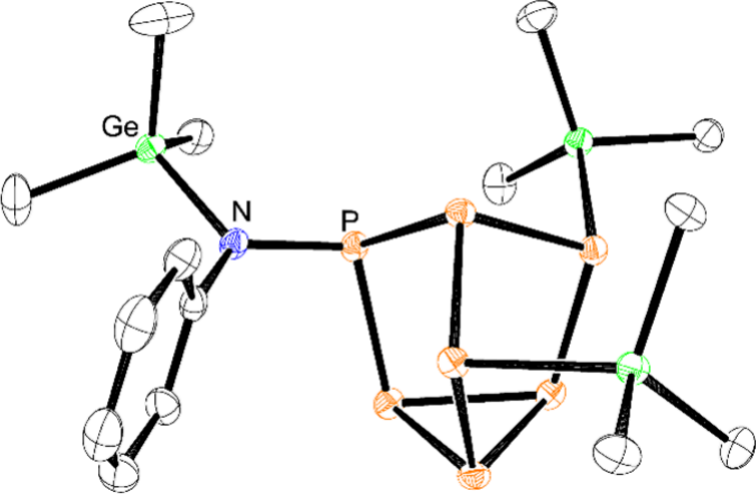
Molecular structure of **6**. Anisotropic displacement ellipsoids pictured at 50% probability. Hydrogen atoms have been omitted for clarity. Color code: phosphorus, orange; germanium, green; nitrogen, blue; carbon, black.

However, when cluster **2** was reacted with 3 equiv of PhN_3_, investigation of the reaction mixture by ^31^P NMR spectroscopy revealed complete consumption of the starting material and 14 new resonances, along with small amounts of cluster decomposition. Subsequent mass spectrometry studies confirmed the presence of the mono-inserted product **6** (CCDC 2351076) and the bis-inserted product **7** (Scheme 2). Single crystals suitable for XRD studies were grown from the mixture of products, and a crystal structure solution further verified the formation of **6** (Figure 5), which was isolated in a 32% yield. From this structural data, the P–N bond length was determined to be 1.683(15) Å, the N–Ge bond length to be 1.885(14) Å, and the P–N–Ge bond angle to be 118.7(8)°. The two remaining Ge–P bonds had an average length of 2.356(5) Å, consistent with the Ge–P bond lengths of the unreacted starting material **2** [2.354(8) Å].^30^^31^P NMR analysis of a solution of crystals of **6** allowed for the assignment of its corresponding seven signals in the NMR spectrum of the reaction mixture, which, in turn, allowed the seven signals corresponding to the bis-inserted product **7** to be assigned. Analysis of the ^31^P NMR spectrum from the reaction mixture revealed the product ratios to be 38% **6**, 39% **7**, and 23% unknown decomposition products (see SI section 2.4).

**Scheme 3:**
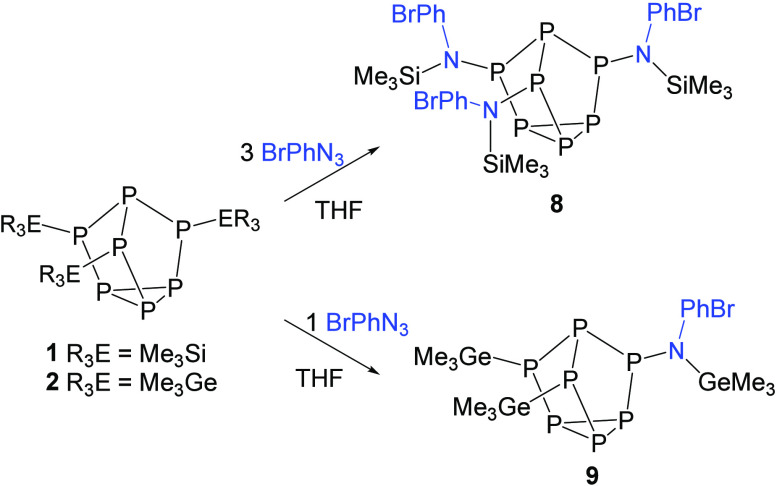
Reactions of 4-Bromophenyl Azide with Clusters **1** and **2**

**Figure 6:**
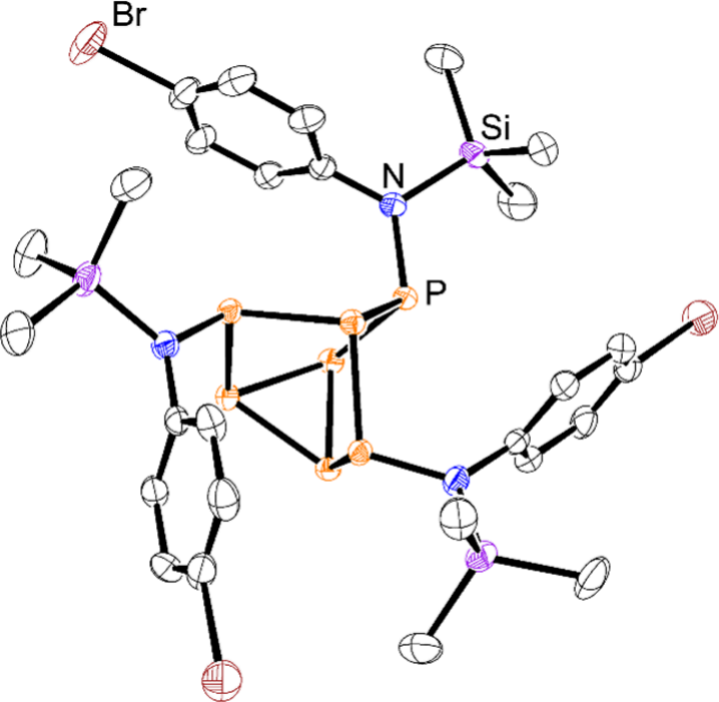
Molecular structure of **8**. Anisotropic displacement ellipsoids pictured at 50% probability. Hydrogen atoms have been omitted for clarity. Color code: phosphorus, orange; silicon, purple; nitrogen, blue; bromine, brown; carbon, black.

Treatment of **1** with 4-BrPhN_3_ enabled isolation of the symmetric tris-inserted product **8** (CCDC 2351077), again confirmed by three characteristic resonances in the ^31^P NMR spectrum and the single resonance at 13.4 ppm in the ^29^Si NMR spectrum with a ^2^*J*_SiP_ coupling constant of 29.8 Hz (Scheme 3). XRD studies further authenticated the identity of **8** (Figure 6), with bond metric data consistent with that previously presented for compound **5**: average P–N and N–Si bond lengths of 1.703(4) Å and 1.774(4) Å, respectively, along with an average P–N–Si bond angle of 115.9(2)°.

In our previously reported isocyanate insertion reactivity with tris-tetrel-functionalized [P_7_] clusters, only the tris-inserted products had been obtained.^13,14^ In fact, when **1** was reacted with 1 equiv of phenyl isocyanate, only the tris-inserted product and unreacted starting material were observed: no mono- or bis-inserted products formed.^14^ To probe whether the amount of organoazide would impact the relative proportions of partially inserted products, clusters **1** and **2** were reacted with varying amounts (1–5 equiv) of benzyl azide, phenyl azide, and 4-bromophenyl azide (data summarized in SI section 3). The reaction mixtures were monitored by ^31^P NMR spectroscopy, and using triphenylphosphine oxide as an internal standard, conversions to the different insertion products were quantified. As the number of equivalents of azide was increased, the cluster product with a greater number of inserted azides was preferred. In the case of the silyl-functionalized cluster **1**, the capture of additional [RN] units and formation of the bis- and tris-inserted products were more facile than those with the germanium-functionalized cluster **2**. For example, when **2** is reacted with 3 or 5 equiv of phenyl azide or 4-bromophenyl azide, the primary products are the bis-inserted clusters. In contrast, when **1** is reacted with the same amounts of these azides, the tris-inserted products are favored, which suggests that silyl-functionalized clusters are more prone to insertion chemistry than the analogous germanium-functionalized systems. This observation is consistent with the reactivity previously reported with isocyanates, where reactions with the silyl-functionalized [P_7_] clusters required less time than those with the germanium derivatives.^13^ However, it is also noteworthy that a mixture of all three insertion products—mono, bis, and tris—was often present in the reaction mixture. In many cases, cluster decomposition could also be observed, and this decomposition increased as the amount of azide was increased. This decomposition is not entirely surprising because azides are oxidizing agents and the [P_7_] cluster framework is prone to decomposition upon oxidation to give soluble and insoluble polyphosphides.

**Figure 7:**
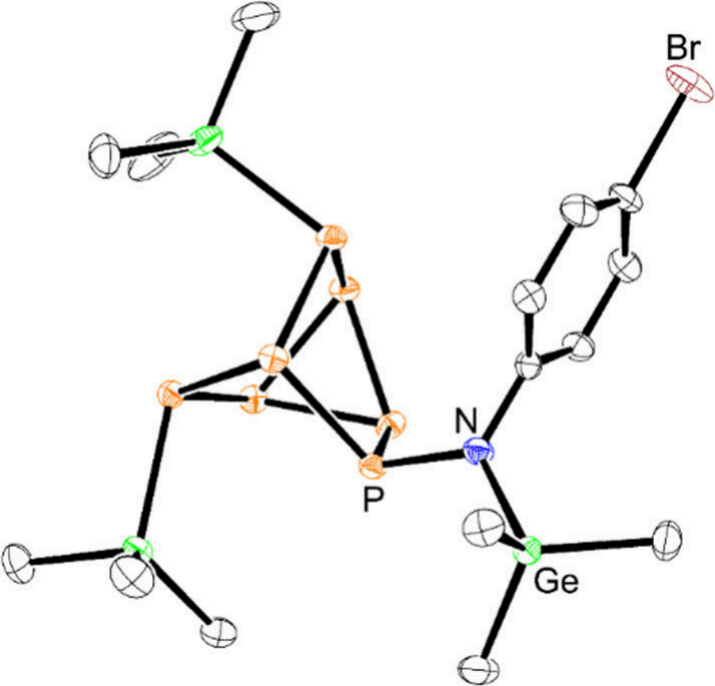
Molecular structure of **9**. Anisotropic displacement ellipsoids pictured at 50% probability. Hydrogen atoms have been omitted for clarity. Color code: phosphorus, orange; germanium, green; nitrogen, blue; bromine, brown; carbon, black.

Although it was not possible to obtain a significant amount of the tris-inserted product from the reaction of **2** with >3 equiv of 4-BrPhN_3_, when **2** was reacted with 1 equiv of 4-BrPhN_3_, the mono-inserted product **9** (CCDC 2351078) was observed in the greatest conversion (Scheme 3). From this reaction mixture, crystals suitable for XRD studies were obtained and further validated the formation of product **9** (Figure 7), with a molecular structure analogous to that of **6**.

### Computational Studies

The electronic structures of the starting clusters and insertion products were computationally investigated to gain further insight into their properties and chemistry. Specifically, we hypothesized that the reaction with an azide to insert an [RN] unit into the P–E bond would partially oxidize the [P_7_] core, which would manifest as a reduction in the electron density attributable to the phosphorus atoms. We also hypothesized that the first insertion would alter the nature of the remaining P–E bonds, which would impact subsequent insertions.

The geometries of the Me_3_E-functionalized clusters **1** and **2** were optimized at the PBE0/6-311G(d,p) level of theory. The computationally optimized structures of the two [P_7_] cores are essentially identical with a root-mean-squared deviation (RMSD) of 0.0030 Å. The geometries of the [BnN] tris-insertion products **3** and **4** were similarly optimized, and, again, the geometries of the [P_7_] cores of the two are nearly identical (RMSD = 0.0063 Å). As observed crystallographically, conversion of **1** to **3** or **2** to **4** results in very little change to the geometry of the [P_7_] core, with the P–P bond lengths only increasing by an average of approximately 1%.

To test our first hypothesis, we conducted an analysis of the topology of the electron densities (ρ) of **1**–**4**. The gradient of the electron density (∇ρ) provides a convenient and natural means of partitioning the total electron density of each molecule between the [P_7_] core and its substituents. The electron density was integrated within the region of space bounded by the P–E (E = Si, Ge, N) surfaces of zero flux and the van der Waals surface of the molecule (Table 1). The insertion of a [BnN] unit into each of the P–Si bonds of **1** to form **3** results in the removal of about three electrons from the core (Table 1). A similar three-electron oxidation of the core occurs upon converting **2** to **4**. Looking at the phosphorus atomic basins individually, one can appreciate that the oxidation of the core stems almost entirely from the loss of an electron from each of the functional-group-bearing phosphorus atoms. Atomic charges (AIM charges) were calculated by subtracting nuclear charges from the integrated electronic charge within each atomic basin. The AIM charges of the functional-group-bearing phosphorus atoms increase by approximately 1 au from **1** to **3** or from **2** to **4** (Table 1). The AIM charges of the other phosphorus atoms are approximately 0 both before and after [BnN] insertion. The oxidation of the functionalized phosphorus atoms was further confirmed with a wavefunction-based natural population analysis (NPA), which shows a similar increase (Table 1). The NPA charges of the remaining phosphorus atoms were again approximately 0 before and after insertion. These results collectively support our hypothesis that the cluster is partially oxidized by reaction with RN_3_ and indicate that the oxidation is localized to the functionalized phosphorus atoms rather than delocalized across the cluster.

**Table 1:** Computational Parameters Describing the Electronic Structures of **1**–**4**

	**1**	**2**	**3**	**4**
[P_7_] ρ integral (e^–^)	106.8	106.0	103.1	103.1
average P–E AIM charge^a^	–0.56	–0.29	0.72	0.74
average P–E NPA charge^a^	–0.23	–0.20	0.46	0.47
P–E bond polarization	63% P	62% P	26% P	26% P
	37% Si	38% Ge	74% N	74% N

a Averaged across equivalent P–E bonds. Values for individual atoms are provided in the Supporting Information.

**Figure 8:**
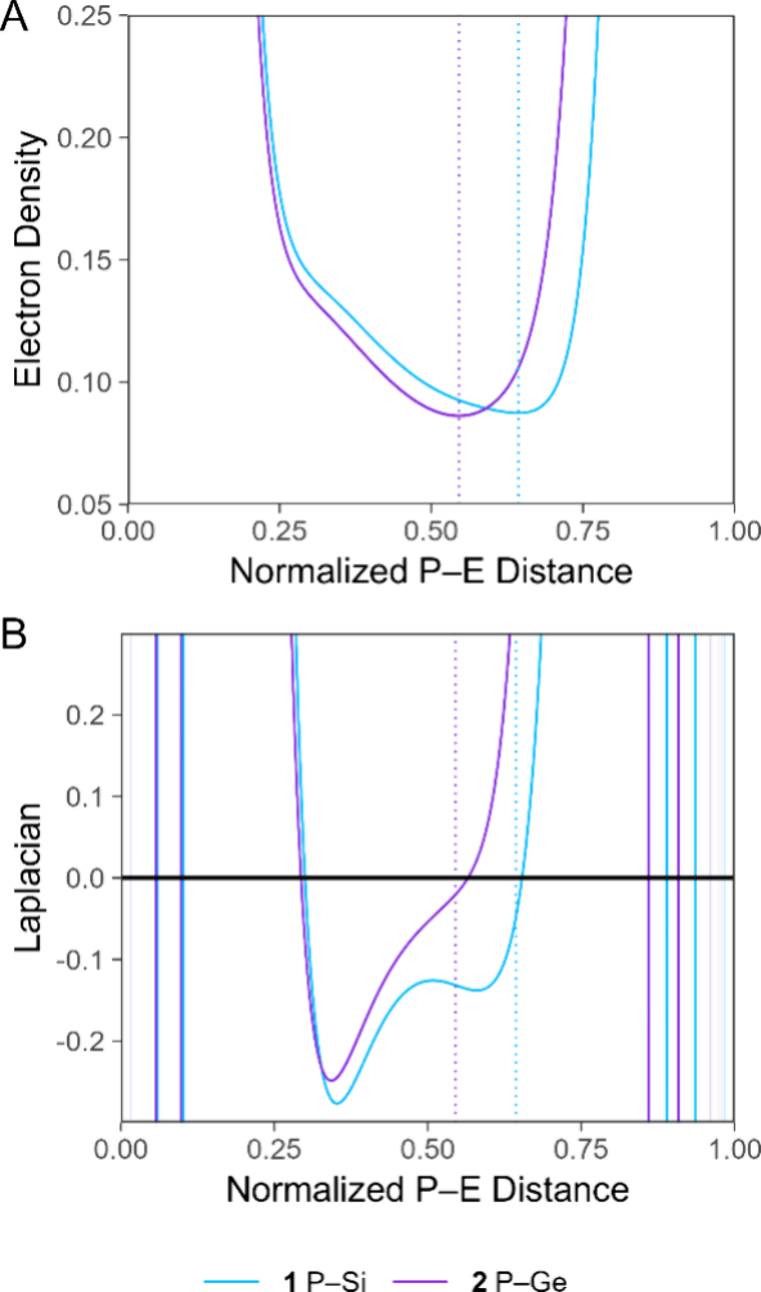
Plots of (A) the electron density (ρ) and (B) the Laplacian of the electron density (∇^2^ρ), both in atomic units, along the indicated P–E interatomic vectors of **1** and **2**. Bond lengths are normalized with the P nucleus at 0 and the E nucleus at 1. The position of the bond critical point is indicated with a color-matched dashed vertical line. Both plots overlay three traces (for the three P–E units) for **1** and **2** each; the curves are virtually indistinguishable and appear as a single trace.

To prepare for an analysis of the effect of [RN] insertion on P–E bonding, we first compared the P–E bonds of **1** and **2**. The topological analysis that permitted quantification of the charge densities of the [P_7_] cores also located bond critical points along each of the three P–E interatomic vectors in both clusters. The average magnitudes of ρ at the bond critical points were nearly identical for **1** and **2** at 0.087 and 0.086 au, respectively. The average values of the Laplacian of ρ (∇^2^ρ) for **1** and **2**, −0.048 au and −0.020 au, respectively, provide a preliminary indication of a greater covalency in the P–Si bond, but it should be noted that both ∇^2^ρ values are very close to zero. It has recently been shown, however, that evaluation of these functions along the full length of the bond path may provide a more accurate representation of polar bonds between heavy elements.^20^ Analysis of ρ along the bond path highlights that both compounds reach a similar minimum value and that the bond critical point lies closer to the tetrel atom in the case of **1** (Figure 8A), as expected based on the variation in the tetrel atom size. The behavior of ∇^2^ρ reveals more telling differences. In the valence region of the P–E bond, **1** features two local minima and a more negative P-proximal minimum (Figure 8B). In contrast, the valence region of **2** features a disappearance of the tetrel-proximal minimum and a smaller phosphorus-proximal minimum. These features are all consistent with the P–Si bond exhibiting a more covalent character.

To better understand the nature of the P–E bonding, we analyzed the molecular orbitals of **1** and **2**. The canonical orbitals of these symmetric cluster compounds exhibit a high degree of delocalization, and so the localized natural bond orbitals were studied. Both the P–Si bond of **1** and the P–Ge bond of **2** are formed from the overlap of approximately sp^3^ hybrid atomic orbitals on both the phosphorus and tetrel atoms. For both atoms in both types of bonds, the constituent hybrid atomic orbitals are slightly enriched in p character compared to a strict sp^3^ orbital. As expected, the P–E bonds are polarized, with the more electronegative phosphorus atom contributing to a greater extent than the tetrel atom (Table 1). The bond polarity is approximately the same for **1** and **2**, which is unsurprising given the similarities in the electronegativities of silicon and germanium. This bond polarization is also consistent with the negative NPA charges on the phosphorus atoms that were described above.

**Figure 9:**
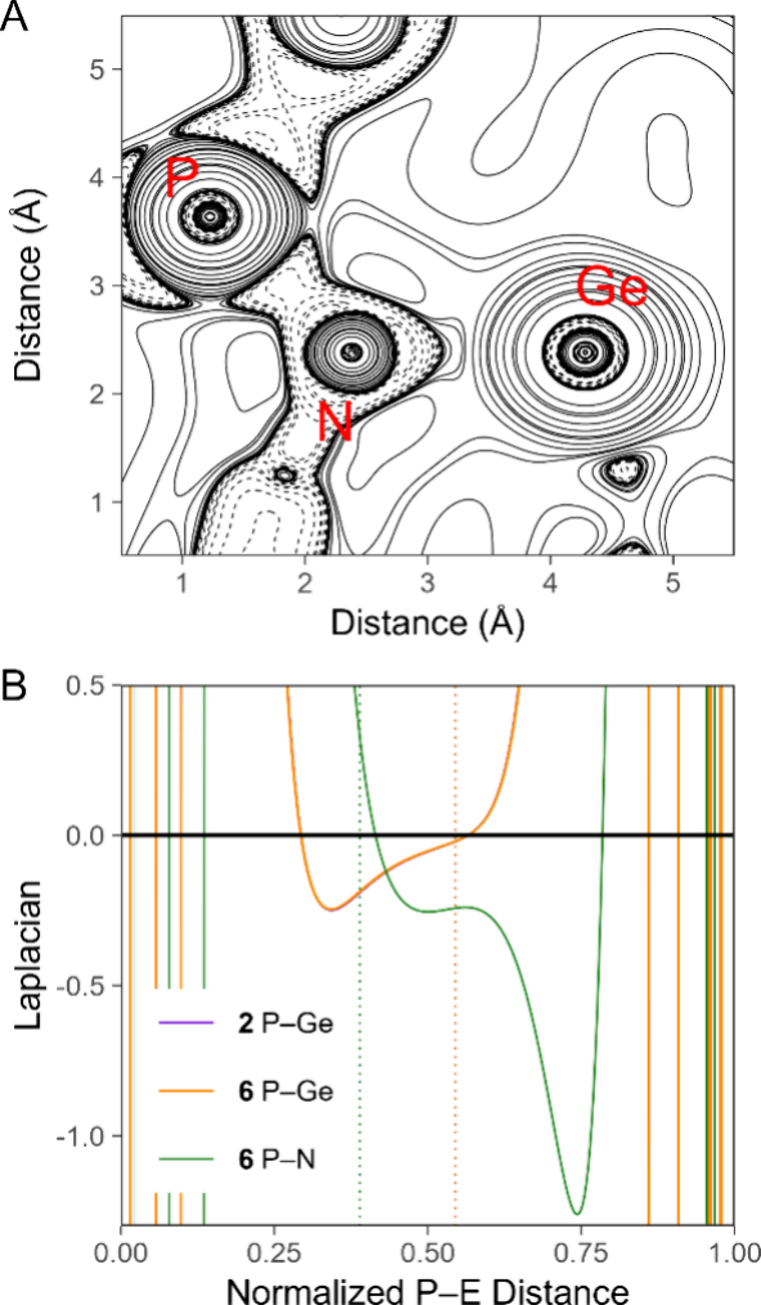
(A) Two-dimensional plot of the Laplacian of the electron density (∇^2^ρ) of **6** in the P–N–Ge plane of the [PhN]-functionalized phosphorus atom. (B) Plots of ∇^2^ρ along the indicated P–E interatomic vectors of **2** and **6**. Bond lengths are normalized with the P nucleus at 0 and the E nucleus at 1. The position of the bond critical point is indicated with a color-matched dashed vertical line. The plot features three distinct P–Ge traces for **2** and two distinct P–Ge traces for **6**, but they overlay nearly perfectly.

To assess whether insertion of an [RN] group into one of the P–E bonds impacts the reactivity of the remaining P–E bonds, we compared the bonding in **2** to that in **6**, the intermediate in the reaction of **2** with PhN_3_. [PhN] insertion, as opposed to [BnN] insertion, was explored here because of our capacity to compare the computationally optimized structure of **6** to the crystallographic data described above for this mono-insertion product. The overall RMSD between the computationally optimized and crystallographic geometries, permitting torsional freedom, was 0.358 Å with a RMSD between the [P_7_] cores of 0.021 Å. A topological analysis of the computed electron density of **6** identified a bond critical point between the phosphorus and nitrogen atoms, and a two-dimensional plot of ∇^2^ρ shows the stereotypical morphology of a covalent P–N bond (Figure 9A). A more nuanced view is provided by a one-dimensional plot of ∇^2^ρ along the bond path (Figure 9B). The stark difference between the P–Ge and P–N bonds is readily apparent, with the P–N bond exhibiting two local minima in the valence region compared the P–Ge bond. Moreover, the magnitude of ∇^2^ρ at the phosphorus-proximal minimum is approximately the same for the P–Ge and P–N bonds in **6**, but the nitrogen-proximal minimum of the P–N bond achieves a much more negative ∇^2^ρ value than the P–Ge bond. This result speaks to the greater covalency of the P–N bond. It can also be seen that the bond critical point lies closer to the phosphorus atom, consistent with the reversal of polarization upon [RN] insertion that was described above.

Last, we compared the three P–Ge bonds of **2** to the two P–Ge bonds of **6** that remain after [PhN] insertion. The plots of ∇^2^ρ for these bonds are essentially identical (Figure 9B). This result suggests that insertion into one of the P–Ge bonds does not drastically impact the nature of the bonding at the remaining two P–Ge bonds. We have previously shown that the bridging phosphorus–tetrel bonds are prone to insertion reactions with heteroallenes. For example, cluster **2** reacts with 3 equiv of phenyl isocyanate to insert the isocyanate molecules into all three of the P–Ge bonds after 4 days.^13^ However, the reaction of cluster **6** with phenyl isocyanate under similar conditions showed no reaction even after 2 weeks, suggesting that reactivity at one site of the phosphorus cluster does impact reactivity at the other sites. In the case of isocyanate insertion, reactivity at the other two sites is suppressed. However, the manner by which “oxidation” at one site of the cluster modulates the behavior at the other sites is yet to be understood and quantified.

## Conclusion

In conclusion, the reactivity of tetrel-functionalized heptaphosphide clusters toward organoazides was probed. It was found that when (Me_3_Si)_3_P_7_ (**1**) and (Me_3_Ge)_3_P_7_ (**2**) were allowed to react with benzyl azide, phenyl azide, and 4-bromophenyl azide, the azide loses N_2_ gas and [RN] units are inserted into the phosphorus–tetrel bonds of the clusters. Insertion into the Si–P bonds of (Me_3_Si)_3_P_7_ (**1**) was found to be more facile than insertion into the Ge–P bonds of (Me_3_Ge)_3_P_7_ (**2**), resulting in the silyl-based cluster favoring the tris-inserted product, while the germanium-based cluster shows a greater preference for the partially inserted (mono and bis) products. Clean isolation of the partially inserted products is interesting because it leaves unreacted sites at the cluster still available for further orthogonal reactivity, and future work is focused on understanding and realizing this potential. The insertion of [RN] units into the phosphorus–tetrel bonds of clusters **1** and **2** yields products where the bridging phosphorus atom of the cluster is directly bonded to nitrogen. Computational investigations were consistent with a ^δ+^P–N^δ−^ polarization of this bond, whereas the starting materials feature P–E bonds with reversed polarity. The analysis also supports the description of the [P_7_] clusters of the [RN] insertion products as oxidized compared to the silyl- or germyl-functionalized starting materials. Oxidation of Zintl polypnictogen clusters often leads to decomposition of the cage structure and a mixture of soluble and insoluble polypnictogens, whereas here a number of clean products are isolated. Although azides have been widely explored in noncluster chemistry to transfer [RN] units, this work represents the first exploration of azide reactivity within Zintl chemistry and expands the profile of small-molecule activations within the field.

## Supporting Information Available

General information, experimental procedures, characterization data, and computational details (PDF)
