# Supplementary material for: Reactivity of Tetrel-Functionalized Heptaphosphane Clusters toward Azides
Source: Inorg Chem. 2024 Jul 16;63(30):13807–14. doi: 10.1021/acs.inorgchem.4c02264 (PMC11289750; doi:10.1021/acs.inorgchem.4c02264)
Supplement: Supplementary file 1 — ic4c02264_si_001.pdf [file ic4c02264_si_001.pdf]

Reactivity of Tetrel Functionalized Heptaphosphane Clusters Towards Azides

Supporting information

William D. Jobbins,[a] Rory T. Cullen,[a] Thomas Stott,[a] Bono van IJzendoorn,[b] Benjamin L. L. Réant,[a] Timothy C. Johnstone,*[c] Meera Mehta*[b]

a. Department of Chemistry, University of Manchester, Oxford Rd, Manchester, M13 9PL.
b. Department of Chemistry, University of Oxford, 12 Mansfield Road, Oxford, OX1 3TA. meera.mehta@chem.ox.ac.uk
c. Department of Chemistry and Biochemistry, University of California Santa Cruz, 1156 High Street, Santa Cruz CA 95064, USA. johnstone@ucsc.edu

Contents

1. Methods and materials 2
1.1. Experimental considerations 2
1.2. Analytical considerations 2
1.3. X-ray diffraction studies 3
1.4. General computational considerations 3
2. Synthesis and Characterization Data for $(R_3E)_3Pn_7$ Azide Inserted Products 3
2.1. Synthesis of $(Me_3Si\text{-}NBn)_3P_7$ (3) 3
2.2. Synthesis of $(Me_3Ge\text{-}NBn)_3P_7$ (4) 8
2.3. Synthesis of $(Me_3Si\text{-}NPh)_3P_7$ (5) 11
2.4. Synthesis $(Me_3Ge\text{-}NPh)(Me_3Ge)_2P_7$ (6) 15
2.5. Synthesis of $(Me_3Si\text{-}NPhBr)_3P_7$ (8) 20
3. Quantification of *mono*-, *bis*- and *tris*-inserted product distributions as azide stiochiometry is tuned 23
3.1. General procedure 23
3.2. Product distributions from the reactions of clusters 1 and 2 with benzyl azide 23
3.3. Product distributions from the reactions of clusters 1 and 2 with phenyl azide 25
3.4. Product distributions from the reactions of clusters 1 and 2 with 4-bromophenyl azide 26
4. Atoms in Molecules calculations 28
5. Optimized structures at the PBE0/6-311G(d,p) level of theory. 29
6. Crystallography tables 46
7. References 49

1. Methods and materials

1.1. Experimental considerations

All manipulations were carried out under a nitrogen atmosphere using standard Schlenk-line and glovebox methodology. All glassware was flame-dried before use.

Innovative Technologies anhydrous engineering solvent purification system was used to obtain dry THF, Et_2O and pentane. Solvents obtained in this way were subsequently degassed. C_6D_6 and THF-d_8 were dried over 3 Å molecular sieves.

Me_3SiCl (Sigma-Aldrich), Me_3GeCl (TCI), benzyl azide (BnN_3) (Sigma-Aldrich), phenyl azide (PhN_3) (Fluorochem), and 4-bromophenyl azide (4-$BrPhN_3$) (Fluorochem) were purchased from their respective vendors and used without further purification. $(Me_3Si)_3P_7$ (**1**) and $(Me_3Ge)_3P_7$ (**2**) were prepared following literature protocols and used in their crystalline form.[1]

1.2. Analytical considerations

NMR spectroscopy – ^1H, $^{13}C\{^1H\}$, ^{29}Si DEPT90, ^{31}P NMR spectra were recorded on a Bruker AVIII 400 spectrometer (operating frequencies: 399.78 MHz, 100.53 MHz, 79.48 MHz and 149.14 MHz for ^1H, ^{13}C, ^{29}Si and ^{31}P respectively).
^1H and ^{13}C chemical shifts were internally referenced to the residual solvent resonances (C_6D_6: ^1H δ = 7.16 ppm, $^{13}C\{^1H\}$ δ = 128.02 ppm, THF-d_8: ^1H δ = 3.58, 1.73 ppm, $^{13}C\{^1H\}$ δ = 67.57, 25.37 ppm. ^{29}Si and ^{31}P chemical shifts were externally referenced to Me_4Si and H_3PO_4 respectively. NMR samples were prepared under an inert nitrogen atmosphere in a 5 mm J Young NMR tube. ^{31}P NMR experiments are not quantitative, and conversion determined using them are approximate and involved the use of an internal standard.

Elemental analysis – Elemental analysis was carried out by the microanalysis service at the University of Manchester using a Flash 2000 elemental analyser. Where elemental analysis data within ±0.5% could not be obtained, best data is provided. Note, that Melen and co-workers have previously found that random error can lead to discrepancies greater than ±0.4%.[2]

Mass spectrometry – Mass spectrometry was carried out by the mass spectrometry service at the University of Manchester using an electrospray ionization equipped Thermo Orbitrap Executive Plus Extended Mass Range mass spectrometer. Mass spectrometry samples were prepared under an inert nitrogen atmosphere and injected directly into the ionization source of the spectrometer.

1.3. X-ray diffraction studies

Data collection: X-ray diffraction (XRD) data was collected for compounds **3**, **4**, **5**, **6**, **8** and **9** on a dual source Rigaku FR-X rotating anode at 100 K with Cu-Kα (1.54184 Å) radiation, equipped with a Hypix000HE detector and Oxford cryosystem. X-ray data was collected using CrysAlisPro software.

Crystal structure determination and refinements: X-ray data was processed and reduced using CrysAlisPro. Absorption correction was performed using empirical methods (SCALE3 ABSPACK) based upon symmetry-equivalent reflections combined with measurements at different azimuthal angles. The crystal structure was solved and refined against all F^2 values using the SHELX and Olex2 suite of programmes.[3] All non-hydrogen atoms were refined anisotropically. Hydrogen atoms were placed in calculated positions and refined using idealized geometries and assigned coupled isotropic displacement parameters.

Crystallographic data have been deposited with the CCDC (CCDC 2351073-2351078).

1.4. General computational considerations

Density Functional Theory (DFT) calculations, including NBO analyses,[4] were performed with the Gaussian 09 program package 3 (version g09, rev.d01).[5] Geometry optimisation frequency calculations and natural bond order analysis were performed at the PBE0/6-311G(d,p) level of theory.[6] No symmetry constraints were applied during optimisation. All minima were confirmed by the absence of imaginary frequencies. Initial geometries were prepared using X-ray diffraction coordinates where available and Facio V22.1.1.64 software. AIM (Atoms in Molecules) analysis were performed using the Multiwfn (version 3.8) software.[7] Basin integrations were performed with a grid spacing of 0.06 a.u. and explicit refinement of basin boundaries.

2. Synthesis and Characterization Data for $(R_3E)_3Pn_7$ Azide Inserted Products

2.1. Synthesis of $(Me_3Si\text{-}NBn)_3P_7$ (3)

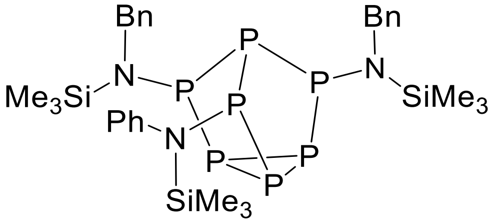

A J Young ampoule was loaded with a stir bar and $(Me_3Si)_3P_7$ **1** (100 mg, 0.23 mmol, 1 eq.), dissolved in THF (2 mL) and cooled to –78 °C. A separate J Young ampoule was loaded with benzyl azide (91 mg, 0.69 mmol, 3 eq.) dissolved in THF (1 mL). The azide solution was added dropwise to the yellow solution of $(Me_3Si)_3P_7$ over a period of 15 min. During addition, the reaction mixture changed colour from pale yellow to a slightly deeper yellow. The reaction was allowed to stir at –78 °C for 1 h and then stirred at room temperature for an additional 12 h. The solvent was removed under reduced pressure to give a yellow oil. This oil was stirred in pentane and re-dried, yielding a yellow waxy solid. This solid was dissolved in Et_2O (2 mL) and slow evaporation of this solution to around half of its original volume yielded block shaped crystals, suitable for single crystal XRD analysis.

Isolated yield: 31 mg, 18%

^{1}H NMR (400 MHz, 298 K, C_6D_6): δ = 7.29 - 7.21 (overlapping signals, 15H, CH_2*Ph*), 1.37 (s, 6H, *CH_2*Ph), 0.01 (s, 27H, *Me*$_3$Si) ppm.

$^{13}C\{^{1}H\}$ NMR (101 MHz, 298 K, C_6D_6): δ = 141.4 (s, *Ph*), 128.7 (s, *Ph*), 127.2 (s, *Ph*), 126.9 (s, *Ph*), 30.2 (s, *CH_2*Ph), 0.7 (d, $^{3}J_{CP}$ = 9.3 Hz, *Me_3*Si) ppm.

^{31}P NMR (162 MHz, 298 K, C_6D_6): δ = 115.8 - 111.2 (m, 3P, *bridging*), –199.9 - –207.4 (q, $^{1}J_{PP}$ = 406.0 Hz, 1P, *apical*), –223.0 - –226.3 (m, 3P, *basal*) ppm.

^{29}Si DEPT90 NMR (79 MHz, 298 K, C_6D_6): δ = 14.9 (d, $^{2}J_{SiP}$ = 27.9 Hz, Me_3*Si*) ppm.

Mass spectrometry (ESI pos/neg): For $C_{30}H_{49}N_3P_7Si_3$ ($[M+H]^+$) Calcd.: 752.1392; found: 752.1396.

Elemental analysis: For $C_{30}H_{48}N_3Si_3P_7$: Calcd.: C 47.93, H 6.44, N 5.59; found: C 45.38, H 6.38, N 5.23.

Note: The elemental analysis data presented is the best attempt out of multiple attempts and from crystalline material.

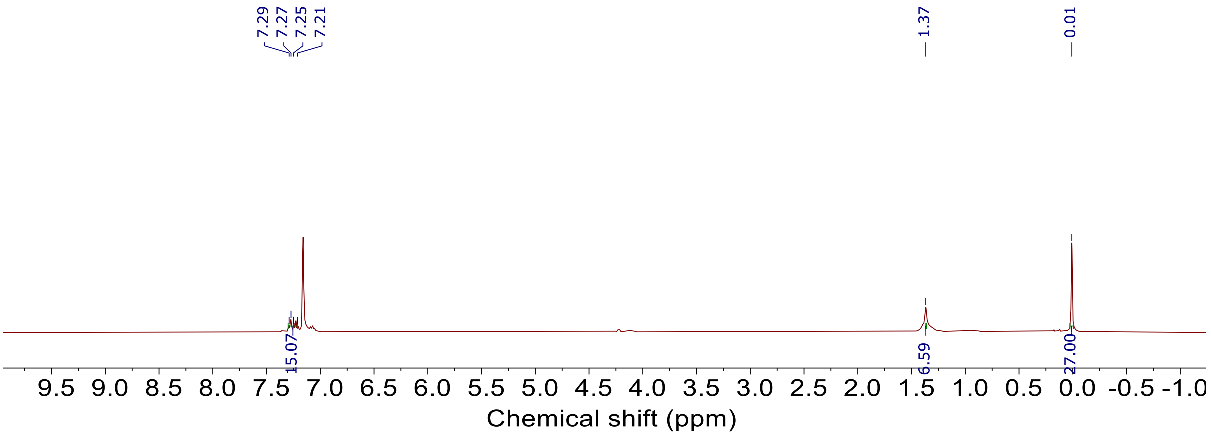

Figure S1. ^{1}H NMR (C_6D_6) spectrum of **3**.

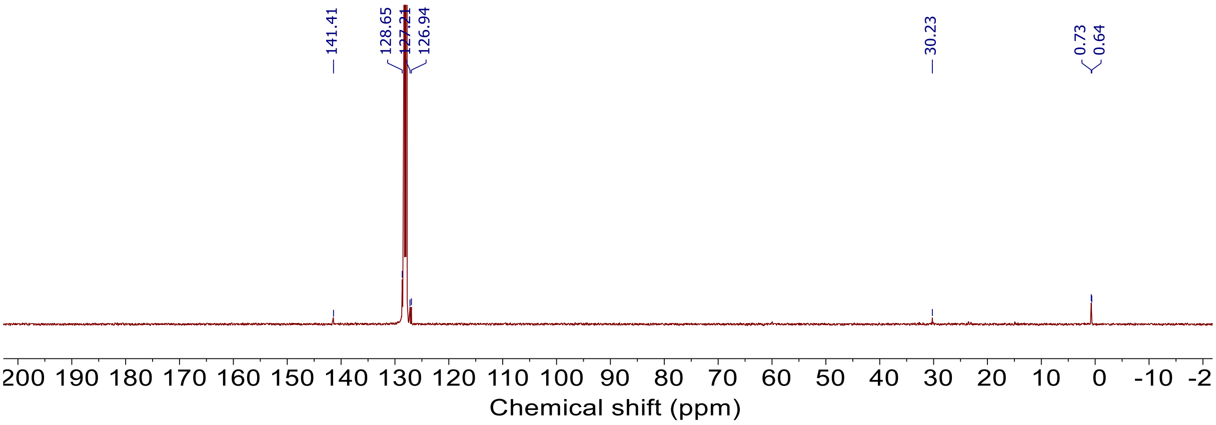

Figure S2. $^{13}C\{^{1}H\}$ NMR (C_6D_6) spectrum of **3**.

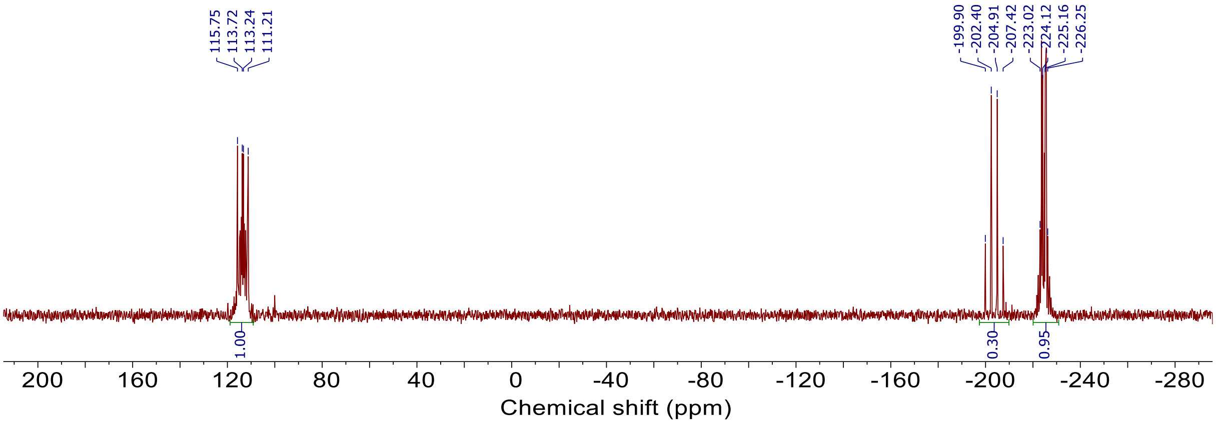

Figure S3. ^{31}P NMR spectrum (C_6D_6) of **3**.

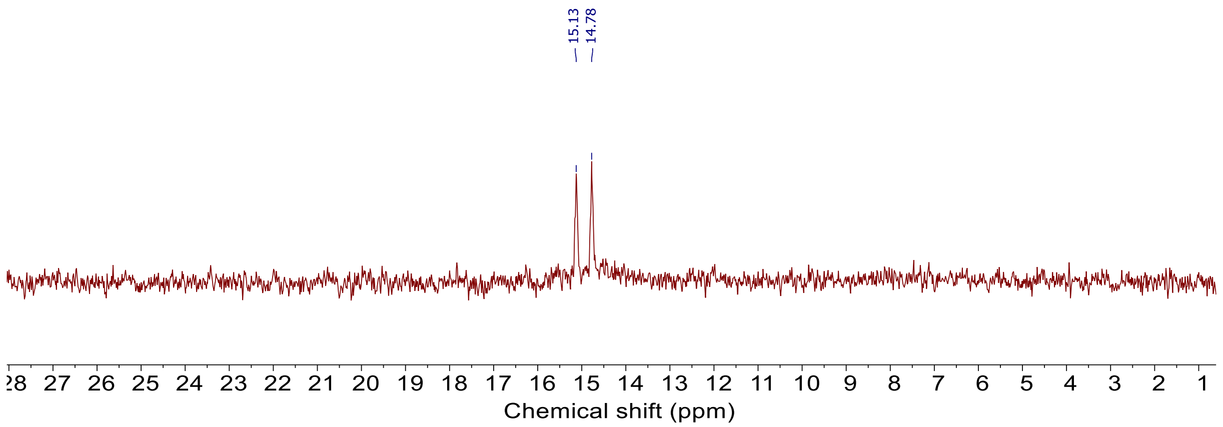

Figure S4. ^{29}Si DEPT90 NMR (C_6D_6) spectrum of **3**.

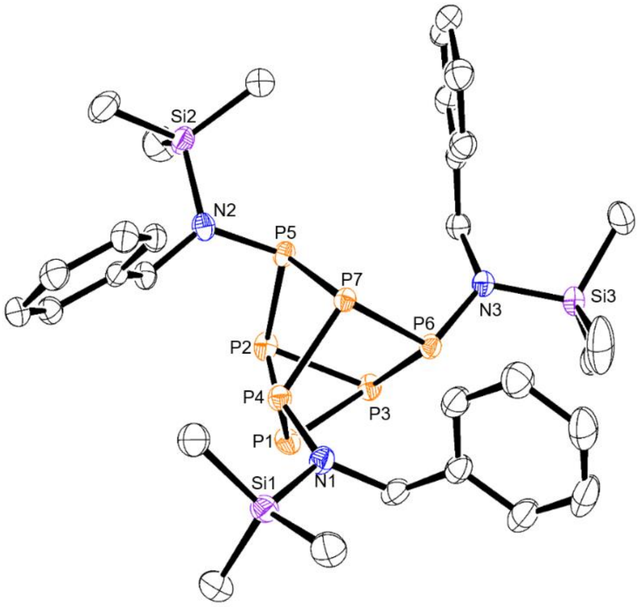

Figure S5. Molecular structure of **3**. Anisotropic displacement ellipsoids captured at 50% probability. Hydrogen atoms have been omitted for clarity. Phosphorus: orange; Nitrogen: blue; Silicon: purple; Carbon: black.

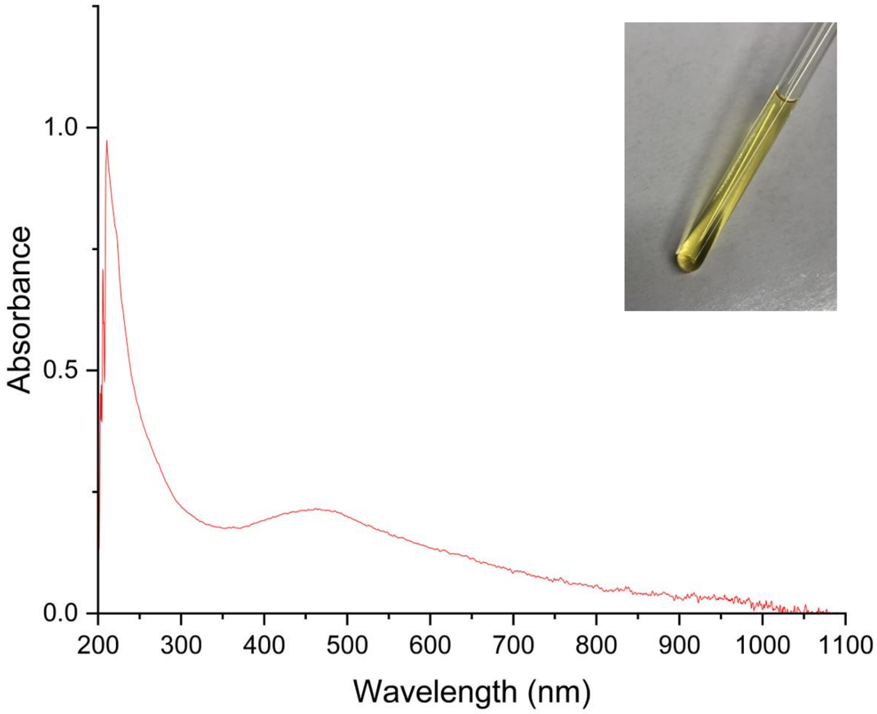

Figure S6. UV-Vis spectrum of $(Me_3Si)_3P_7$ (0.03 mM in THF).

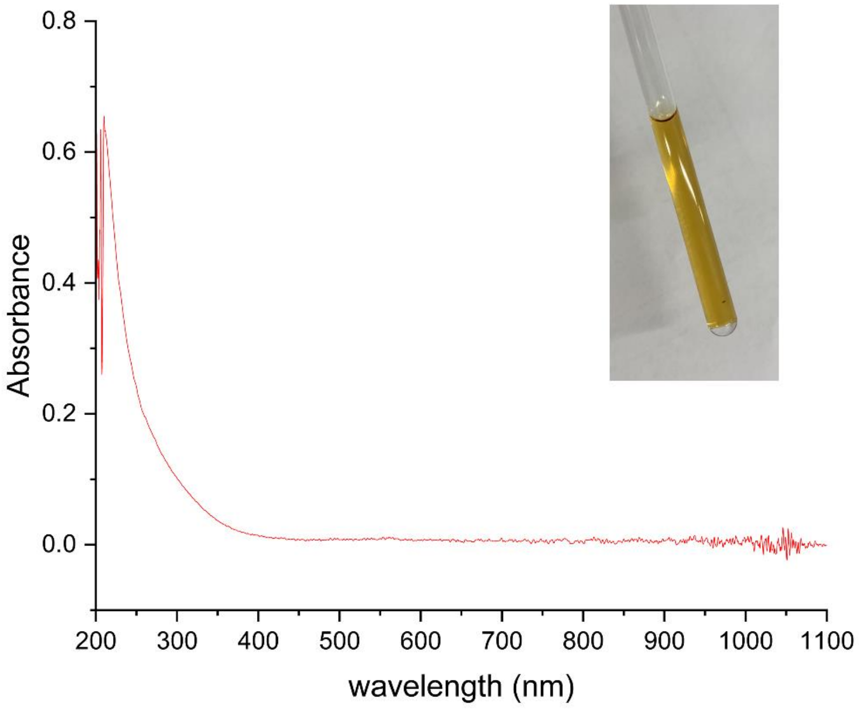

Figure S7. UV-Vis spectrum of **3** (0.03 mM in THF).

2.2. Synthesis of $(Me_3Ge\text{-}NBn)_3P_7$ (4)

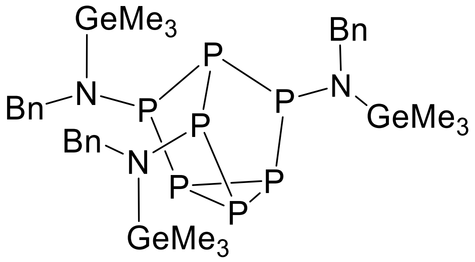

A J Young ampoule was loaded with a stir bar and $(Me_3Ge)_3P_7$ **3** (100 mg, 0.18 mmol, 1 eq.), dissolved in THF (2 mL) and cooled to –78 °C. In a separate J Young ampoule, benzyl azide (70 mg, 0.53 mmol, 3 eq.) was dissolved in THF (1 mL). The azide solution was added dropwise to the pale yellow THF solution of $(Me_3Ge)_3P_7$ over a 15 min period. The reaction mixture turned a slightly darker yellow with the addition of benzyl azide. The reaction was allowed to stir for 1 h at –78 °C and allowed to warm to room temperature. The reaction was then stirred for an additional 2 days at room temperature. Then the solvent was removed under reduced pressure, yielding a yellow oily solid. This residue was dissolved in Et_2O (2 mL) and slow evaporation of this solution to around half its original volume yielded minimal yellow block shaped crystals, suitable for single crystal XRD analysis.

Isolated yield: 23 mg, 15%

^{1}H NMR (400 MHz, 298 K, THF-d_8): δ = 7.34 - 7.22 (overlapping signals, 15H, *Ph*), 0.35 (overlapping singlets, 6H, *CH_2*Ph), 0.19 (27H, *Me*$_3$Ge) ppm.

^{13}C{^{1}H} NMR (101 MHz, 298 K, THF-d_8): δ = 143.8 (s, *Ph*), 129.8 (s, *Ph*), 128.6 (s, *Ph*), 128.0 (s, *Ph*), 15.8 (s, *C*H$_2$Ph), 1.7 (s, *Me$_3$*Ge) ppm.

^{31}P NMR (162 MHz, 298 K, THF-d_8): δ = 123.7 – 119.2 (m, 3P, *bridging*), –197.9 - –205.4 (q, $^{1}J_{PP}$ = 406.1 Hz, 1P, *apical*), –230.0 - –231.5 (m, 3P, *basal*) ppm.

Mass spectrometry (ESI pos/neg): For $C_{30}H_{49}Ge_3N_3P_7$ ($[M+H]^+$) Calcd.: 889.9725; found: 889.9744.

Elemental analysis: For $C_{30}H_{48}Ge_3N_3P_7$: Calcd.: C 40.69, H 5.46, N 4.75; found: C 40.24, H 5.67, N 3.85.

Note: The elemental analysis data presented is the best attempt out of multiple attempts and highly crystalline material was necessary to obtain this data.

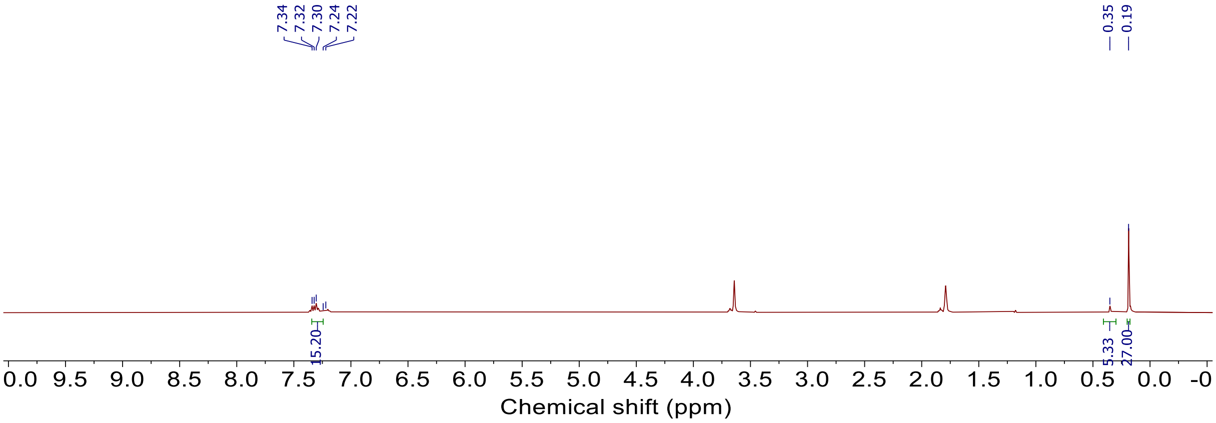

Figure S8. ^{1}H NMR spectrum (THF-d_8) of **4**.

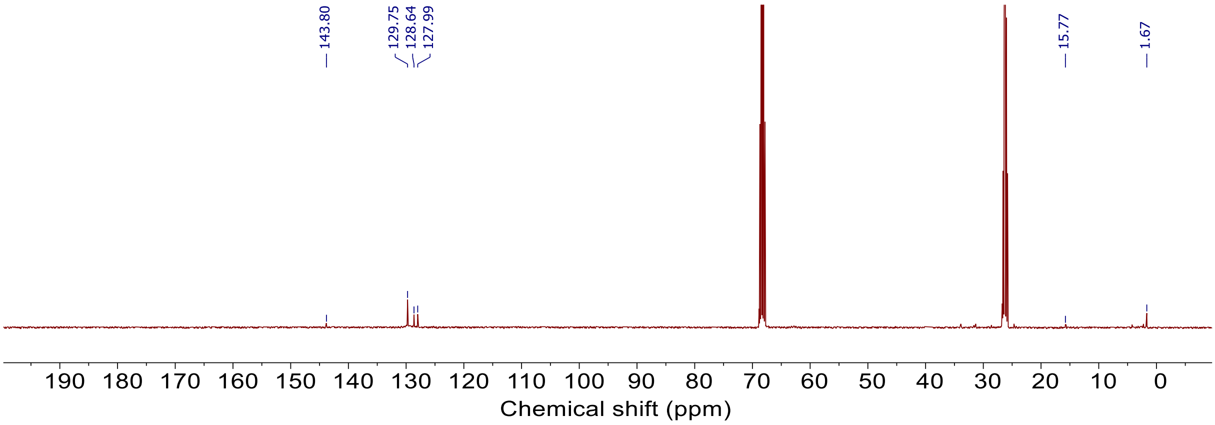

Figure S9. $^{13}C\{^{1}H\}$ NMR spectrum (THF-d_8) of **4**.

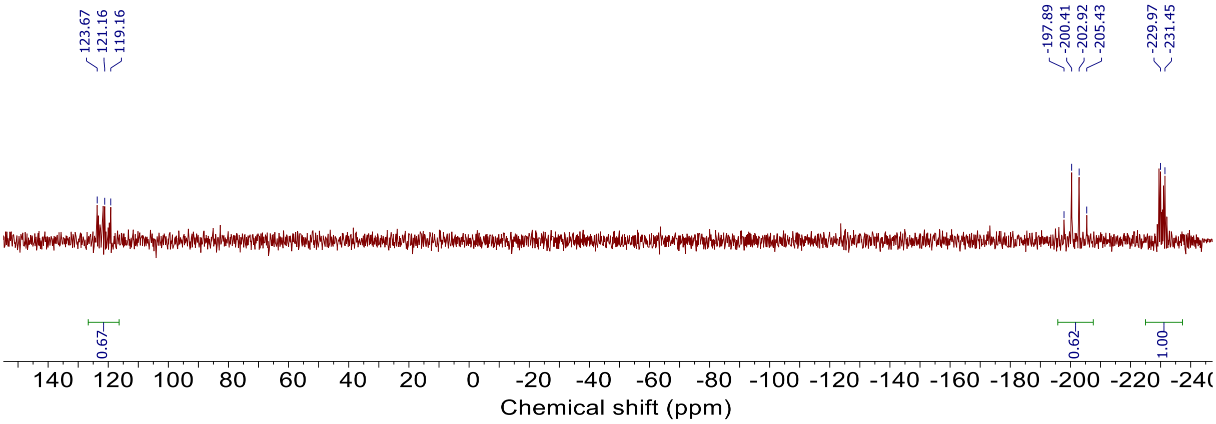

Figure S10. ^{31}P NMR spectrum (THF-d_8) of **4**.

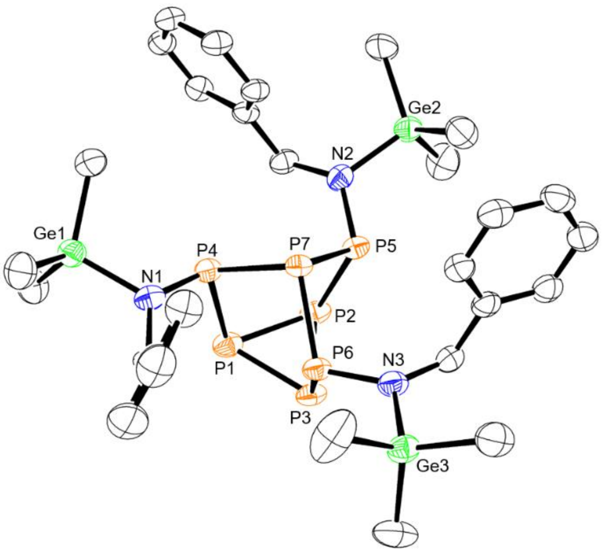

Figure S11. Molecular structure of **4**. Anisotropic displacement ellipsoids captured at 50% probability. Hydrogen atoms have been omitted for clarity. Phosphorus: orange; Nitrogen: blue; Germanium: green; Carbon: black.

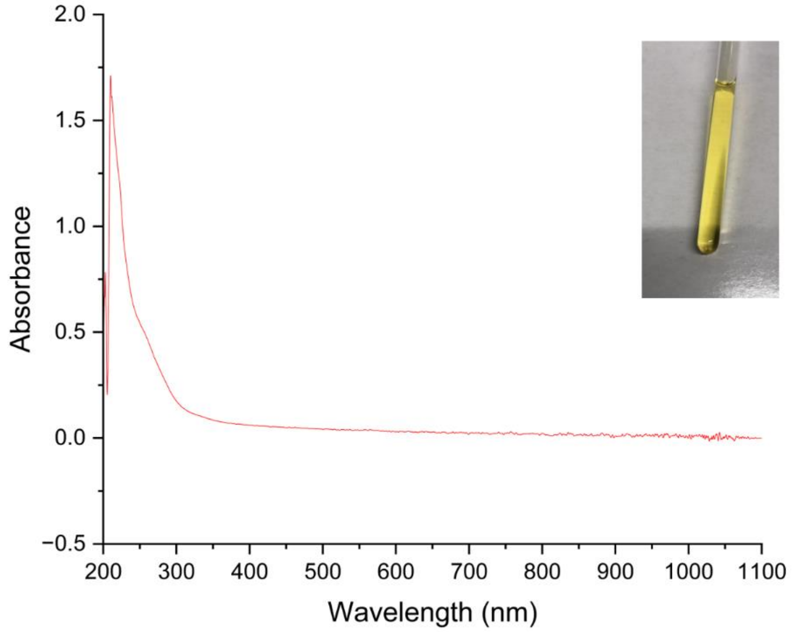

Figure S12. UV-Vis spectrum of $(Me_3Ge)_3P_7$ (0.03 mM in THF).

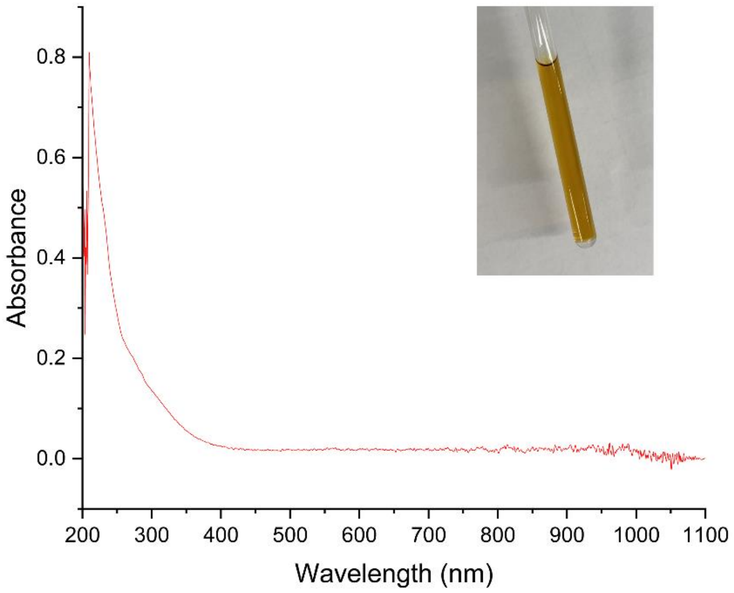

Figure S13. UV-Vis spectrum of **4** (0.03 mM in THF).

2.3. Synthesis of $(Me_3Si\text{-}NPh)_3P_7$ (5)

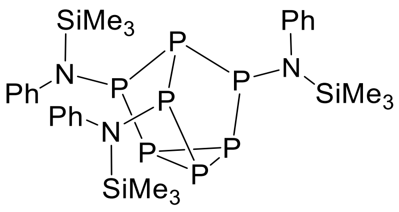

A J Young ampoule was loaded with a stir bar and $(Me_3Si)_3P_7$ **1** (100 mg, 0.23 mmol, 1 eq.), dissolved in THF (2 mL) and cooled to –78 °C. In a separate J Young ampoule, phenyl azide (83 mg, 0.69 mmol, 3 eq.) was dissolved in THF (1 mL). The azide solution was added dropwise to the light yellow THF solution of $(Me_3Si)_3P_7$ over a 15 min period. The reaction was allowed to stir for 1 h at –78 °C, by which point, the solution had turned a darker yellow. The reaction was then stirred for an additional 12 h at room temperature. The solvent was removed under reduced pressure yielding a yellow glassy solid. The residue was dissolved in Et_2O (2 mL) and needle shaped crystals suitable for single crystal XRD analysis were obtained through the cooling of this solution to –35 °C.

Isolated yield: 75 mg, 47%

^{1}H NMR (400 MHz, 298 K, C_6D_6): δ = 7.08 - 6.97 (overlapping signals, 15H, *Ph*), 0.05 (s, 27H, *Me$_3$*Si) ppm.

$^{13}C\{^1H\}$ NMR (101 MHz, 298 K, C_6D_6): δ = 150.1 (br s, *Ph*), 129.3 (s, *Ph*), 129.2 (s, *Ph*), 126.2 (s, *Ph*), 1.0 (d, $^3J_{CP}$ = 7.8 Hz, *Me*$_3$Si) ppm.

^{31}P NMR (162 MHz, 298 K, C_6D_6): δ = 120.5 - 116.1 (m, 3P, *bridging*), –193.9 - –201.5 (qq $^1J_{pp}$ = 406.1 Hz $^2J_{PP}$ = 6.8 Hz, 1P, *apical*), –214.8 - –218.9 (m, 3P, *basal*) ppm.

^{29}Si DEPT90 NMR (79 MHz, 298 K, C_6D_6): δ = 12.6 (d, $^2J_{SiP}$ = 30.1 Hz, Me$_3$*Si*) ppm.

Mass spectrometry (ESI pos/neg): For $C_{27}H_{43}N_3P_7Si_3$ ($[M+H]^+$) Calcd.: 710.0950; found: 710.0963

Elemental analysis: For $C_{27}H_{42}N_3P_7Si_3$: Calcd.: C 45.69, H 5.97, N 5.92; found: C 46.00, H 6.02, N 6.15.

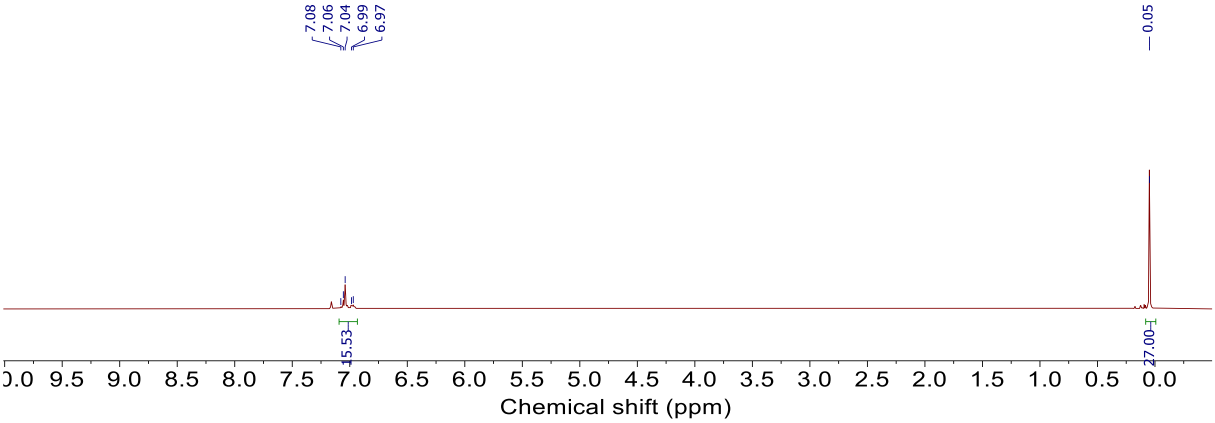

Figure S14. ^1H NMR spectrum (C_6D_6) of **5.**

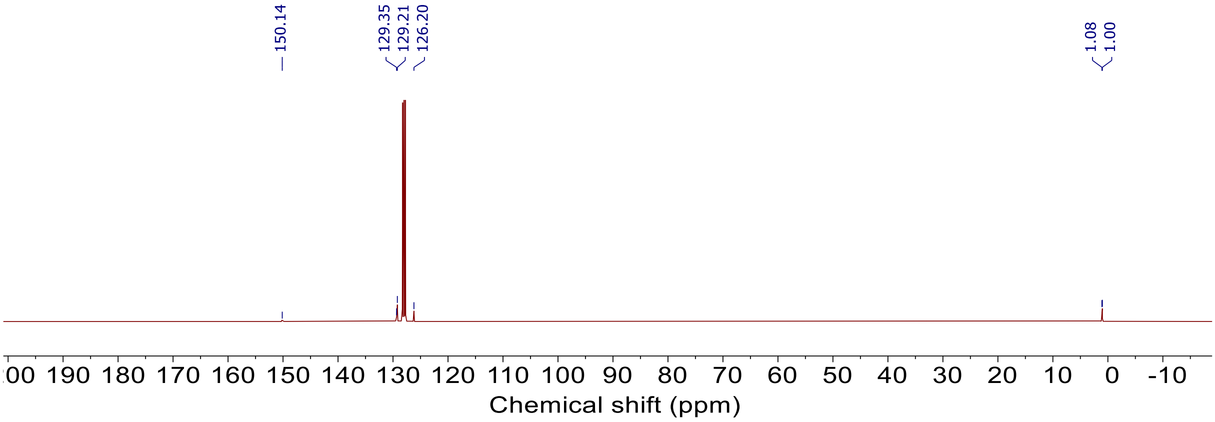

Figure S15. $^{13}C\{^1H\}$ NMR spectrum (C_6D_6) of **5**.

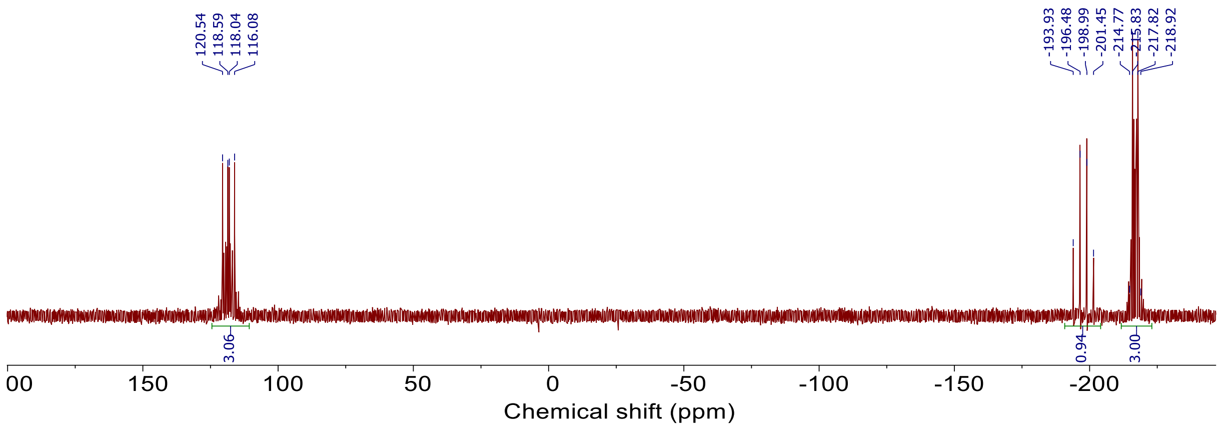

Figure S16. ^{31}P NMR spectrum (C_6D_6) of **5**.

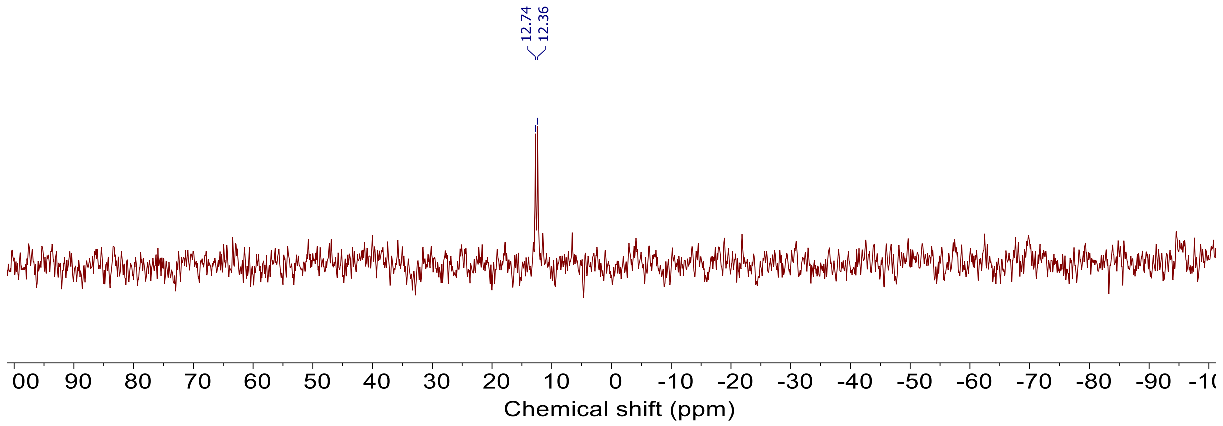

Figure S17. ^{29}Si DEPT90 spectrum (C_6D_6) of **5**.

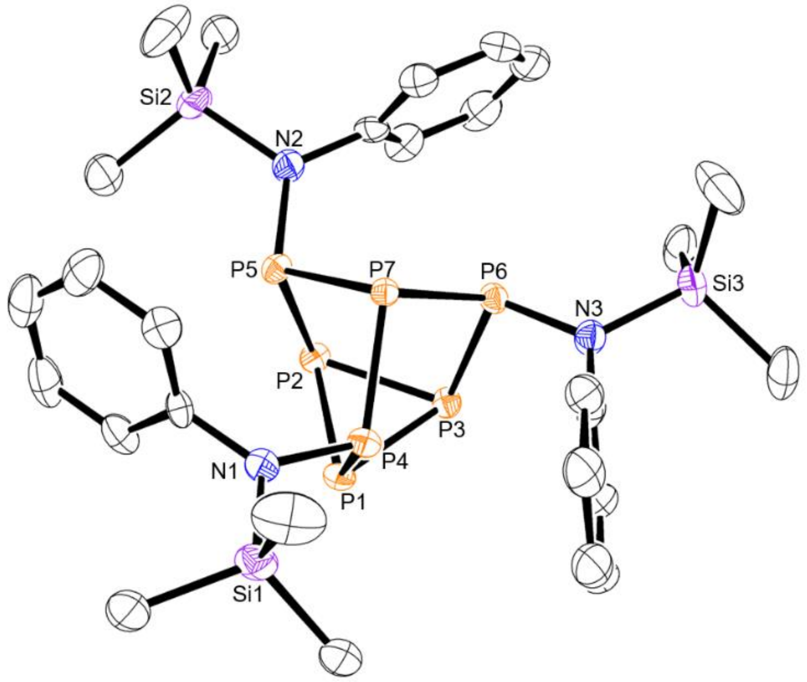

Figure S18. Molecular structure of **5**. Anisotropic displacement ellipsoids captured at 50% probability. Hydrogen atoms have been omitted for clarity. Phosphorus: orange; Nitrogen: blue; Silicon: purple; Carbon: black.

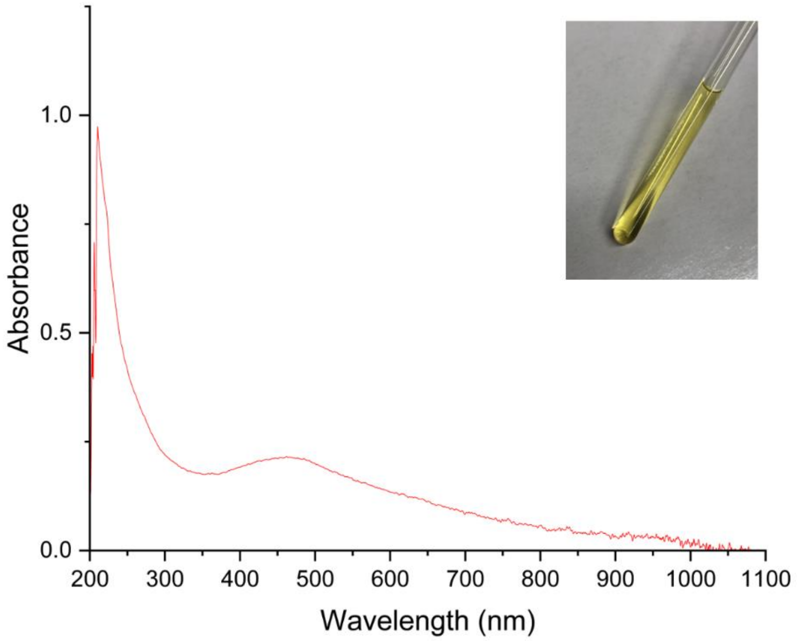

Figure S19. UV-Vis spectrum of $(Me_3Si)_3P_7$ (0.03 mM in THF).

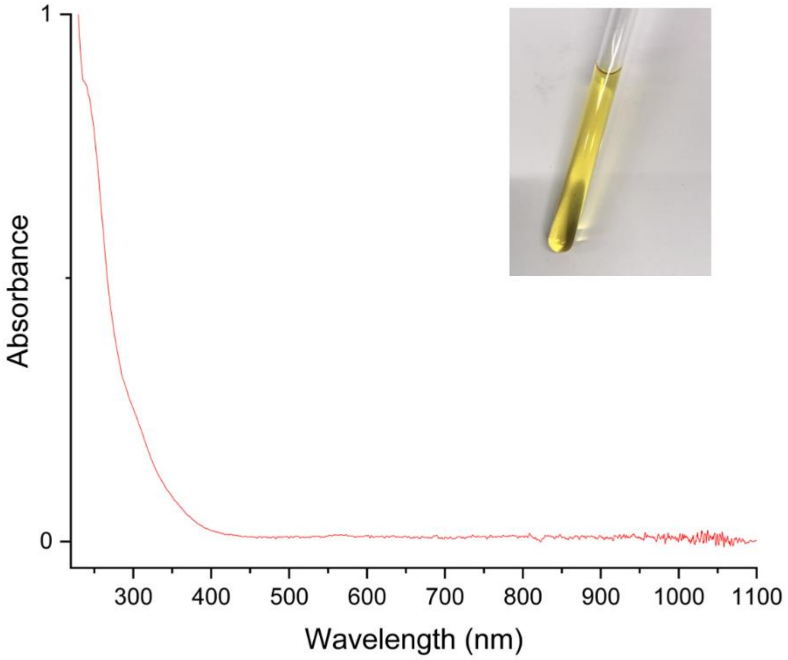

Figure S20. UV-Vis spectrum of **5** (0.03 mM in THF).

2.4. Synthesis $(Me_3Ge\text{-}NPh)(Me_3Ge)_2P_7$ (6)

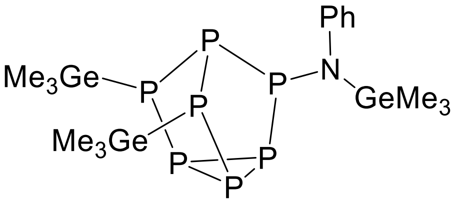

A J Young ampoule was loaded with a stir bar and $(Me_3Ge)_3P_7$ **1** (100 mg, 0.18 mmol, 1 eq.), dissolved in THF (2 mL) and cooled to –78 °C. In a separate J Young ampoule, phenyl azide (63 mg, 0.53 mmol, 3 eq.) was dissolved in THF (1 mL). The azide solution was added dropwise to the yellow/green THF solution of $(Me_3Ge)_3P_7$ over a 15 min period. The reaction was allowed to stir for 1 h at –78 °C, by which point, no colour change from the original solution was observed. The reaction was then stirred for 2 days at room temperature, and then the solvent removed under reduced pressure yielding a yellow waxy solid. The residue was dissolved in Et_2O (2 mL), and crystals suitable for XRD studies obtained through slow evaporation of this solution to around half its original volume and subsequent cooling to –35 ^{o}C.

The mother liquor of the crystallization solution was analyzed and is suspected to contain the *bis*-inserted product, also detected by mass spectrometry. Isolation of this compound was not possible due to the similar solubilities of the *mono*- and *bis*- inserted products. The crude reaction mixture was found to contain 38% of **6** and 39% of **7** with the remaining 23% being unknown decomposition products.

Isolated yield: 38 mg, 32%

^{1}H NMR (400 MHz, 298 K, C_6D_6): δ = 7.01 - 6.89 (overlapping signals, 5H, *Ph*), 0.65 (d, $^{3}J_{HP}$ = 3.6 Hz, 9H, (*Me*$_3$Ge)$_2$(Me$_3$Ge-NPh)P$_7$), 0.32 (d, $^{3}J_{HP}$ = 3.6 Hz, 9H, (*Me*$_3$Ge)$_2$(Me$_3$Ge-NPh)P$_7$), 0.25 (s, 9H, (Me$_3$Ge)$_2$(*Me*$_3$Ge-NPh)P$_7$) ppm.

^{13}C{^{1}H} NMR (101 MHz, 298 K, C_6D_6): δ = 130.1 (s, *Ph*), 128.8 (s, *Ph*), 127.0 (s, *Ph*), 125.3 (s, *Ph*), 5.2 (s, (*Me*$_3$Ge)$_2$(Me$_3$Ge-NPh)P$_7$), 4.4 (s, (*Me*$_3$Ge)$_2$(Me$_3$Ge-NPh)P$_7$), 0.3 (s, (Me$_3$Ge)$_2$(*Me*$_3$Ge-NPh)P$_7$) ppm.

^{31}P NMR (162 MHz, 298 K, C_6D_6): δ = 166.5 - 162.0 (t, $^{1}J_{PP}$ = 374.8 Hz, 1P, *bridging*), 15.6 - 11.5 (t, $^{1}J_{PP}$ = 333.1 Hz, 1P, *bridging*), –75.2 - –79.2 (t, $^{1}J_{PP}$ = 331.0 Hz, 1P, *bridging*), –120.5 - –127.0 (qt, $^{1}J_{PP}$ = 357.8 Hz, $^{2}J_{PP}$ = 53.5 Hz, 1P, *apical*), –141.1 - –146.5 (m, 1P, *basal*), –165.3 - –170.5 (m, 1P, *basal*), –233.1 - –238.8 (m, 1P, *basal*) ppm.

Mass spectrometry (ESI pos/neg): For $C_{15}H_{33}Ge_3NP_7$ ([**6**+H]$^+$;[M+H]$^+$) Calcd.: 661.8437; found: 661.8462.
For $C_{21}H_{38}Ge_3N_2P_7$ ([**7**+H]$^+$;[M+H]$^+$) Calcd.: 752.8860; found: 752.8886.

Elemental analysis: For $C_{15}H_{32}Ge_3NP_7$: Calcd.: C 27.25, H 4.88, N 2.12; found: C 27.97, H 4.98, N 2.47.

*Note: The mass spectrometry data was recorded of the crude reaction mixture of this reaction and shows a mixture of the mono-inserted product **6** and the bis-inserted product **7**.*

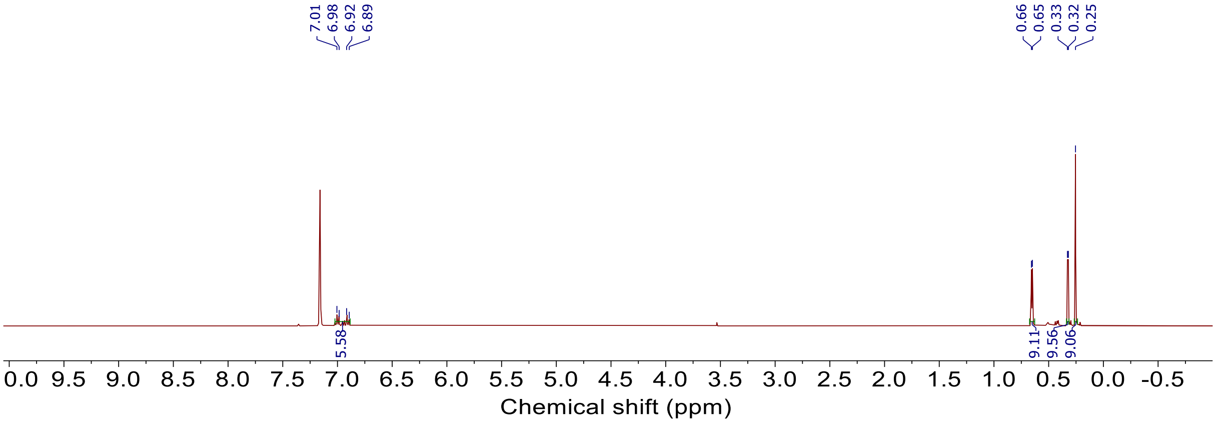

Figure S21. ^{1}H NMR spectrum (C_6D_6) of **6.**

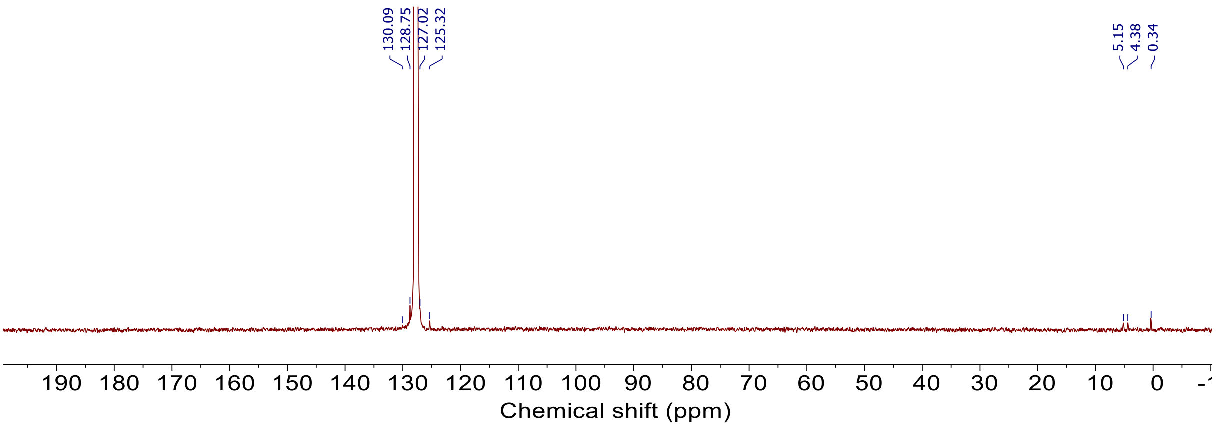

Figure S22. $^{13}C\{^{1}H\}$ NMR spectrum (C_6D_6) of **6**.

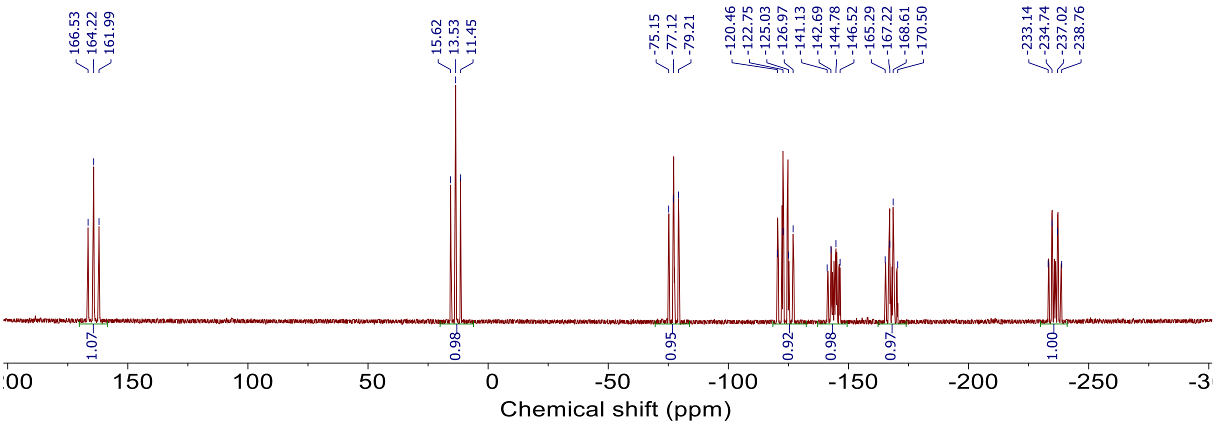

Figure S23. ^{31}P NMR spectrum (C_6D_6) of **6.**

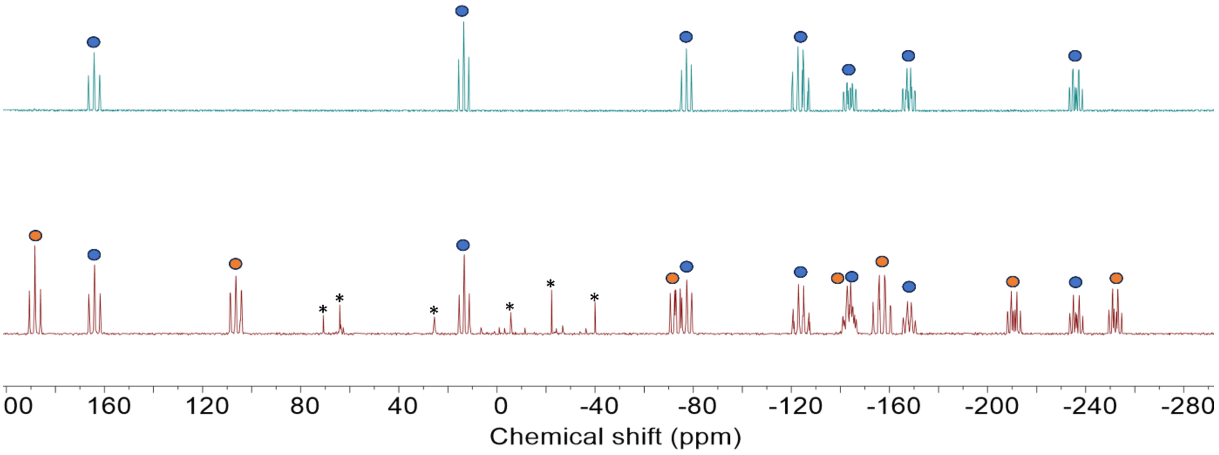

Figure S24. Stacked ^{31}P NMR spectrum (C_6D_6) of **6** (top) and its crude reaction mixture (bottom). Species labelled as *mono-*: blue; *bis-*: orange; decomposition: *.

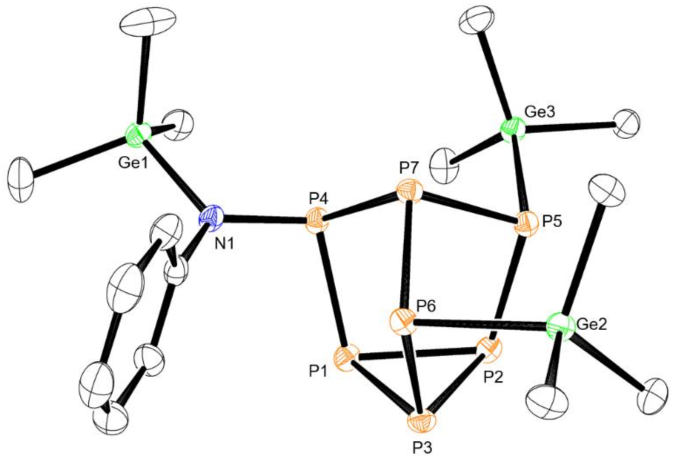

Figure S25. Molecular structure of **6**. Anisotropic displacement ellipsoids captured at 50% probability. Hydrogen atoms have been omitted for clarity. Phosphorus: orange; Nitrogen: blue; Germanium: green; Carbon: black.

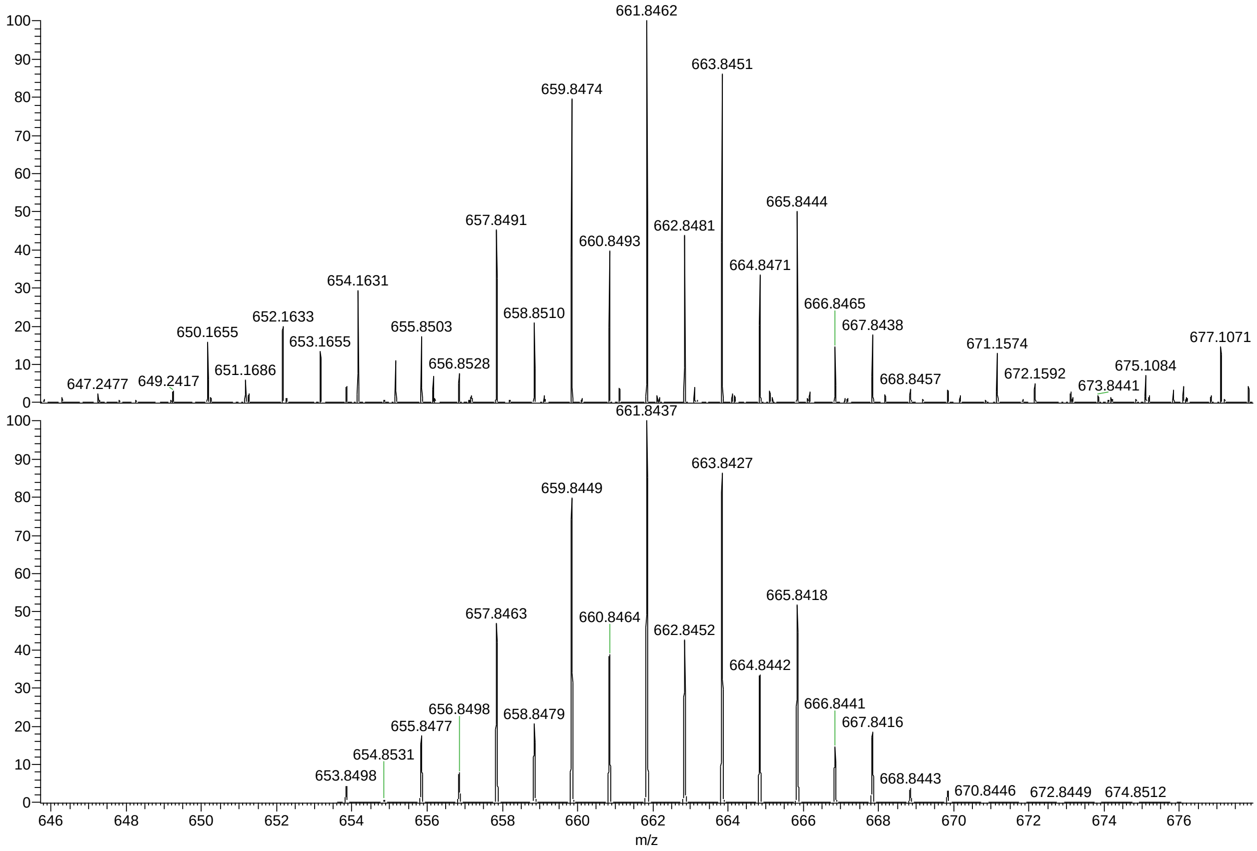

Figure S26. Observed mass spectrum (top) and calculated mass spectrum (bottom) of **6**.

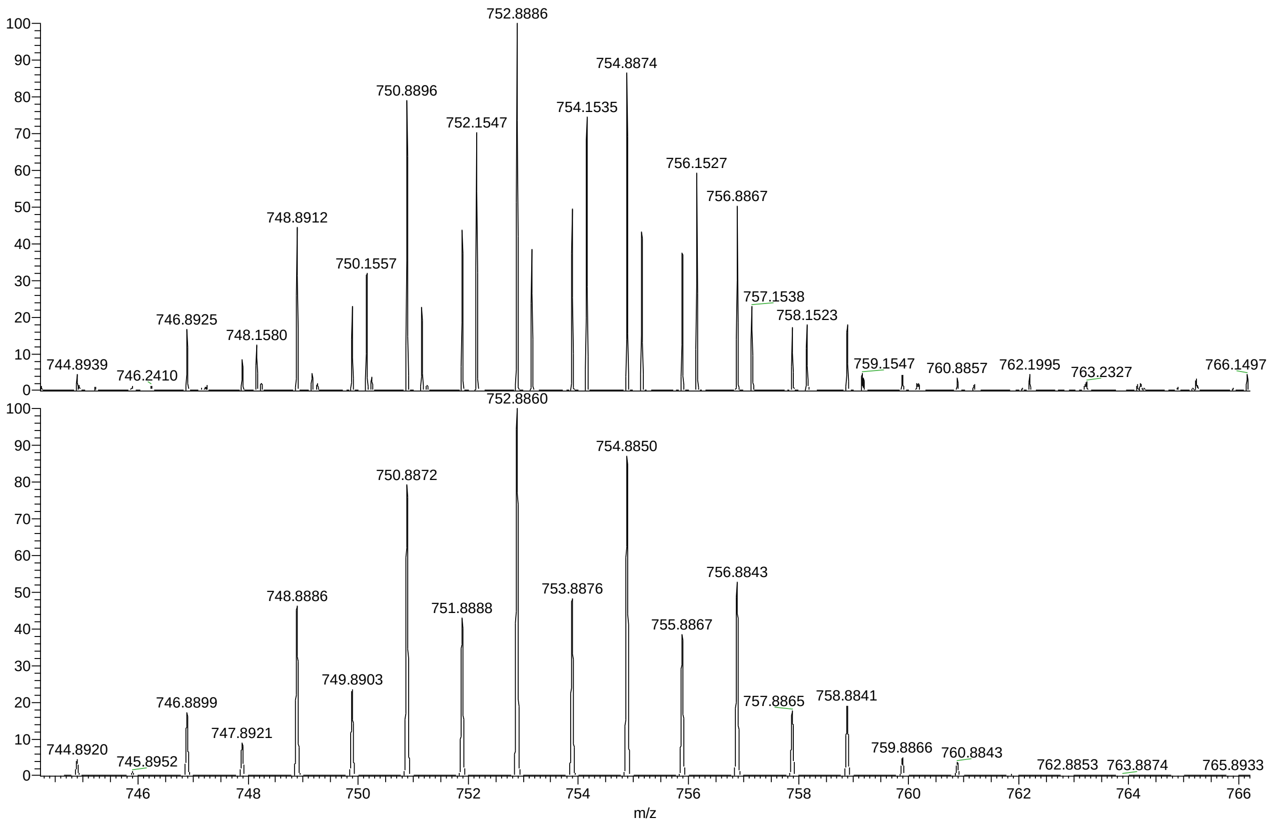

Figure S27. Observed mass spectrum (top) and calculated mass spectrum (bottom) of **7** from the crude reaction mixture.

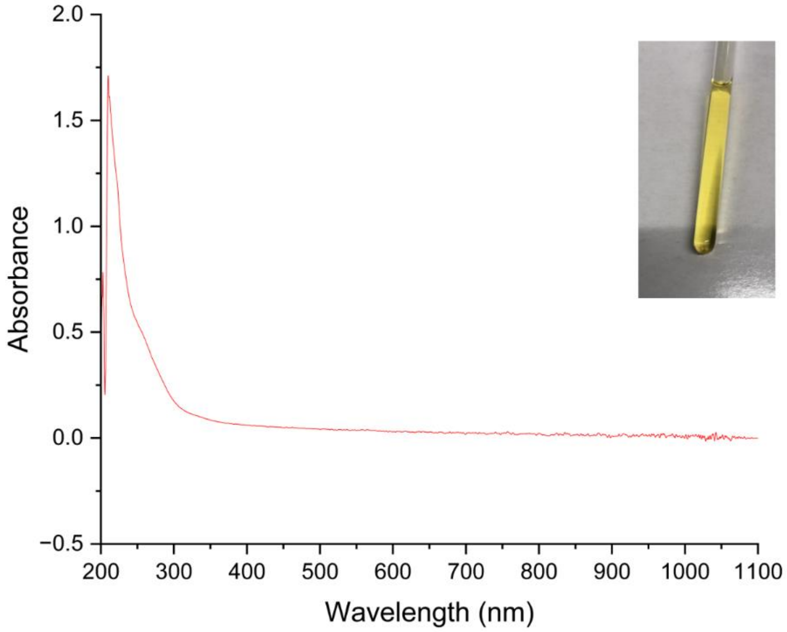

Figure S28. UV-Vis spectrum of $(Me_3Ge)_3P_7$ (0.03 mM in THF).

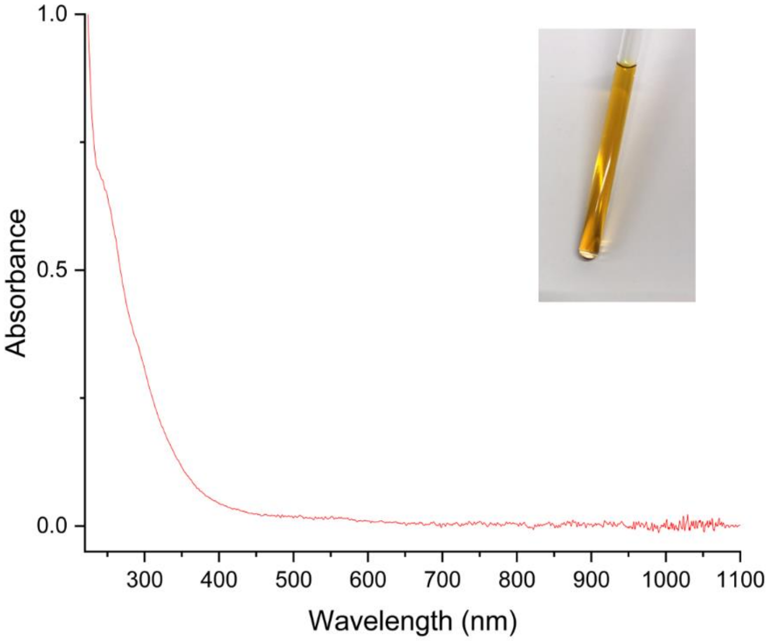

Figure S29. UV-vis spectrum of the reaction mixture for the reaction of **2** with phenyl azide (0.03 mM in THF).

2.5. Synthesis of $(Me_3Si\text{-}NPhBr)_3P_7$ (8)

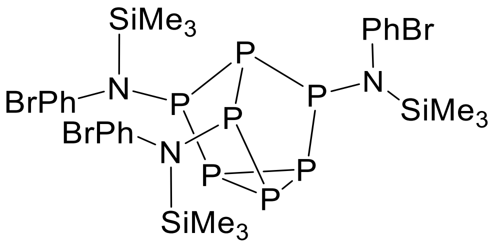

A J Young ampoule was loaded with a stir bar and $(Me_3Si)_3P_7$ **1** (50 mg, 0.10 mmol, 1 eq.), dissolved in THF (2 mL) and cooled to –78 °C. In a separate J Young ampoule, 4-bromophenyl azide (66 mg, 0.34 mmol, 3 eq.) was dissolved in THF (1 mL). The azide solution was added dropwise to the THF solution of $(Me_3Si)_3P_7$ over a 15 min period, over which time no colour change was observed from the original yellow/green solution. The reaction was allowed to stir for an additional 2 days at room temperature. Then the solvent was removed under reduced pressure, yielding a yellow waxy solid. The residue was dissolved in Et_2O (1 mL). Cooling of this solution to –35 ^{o}C yielded large needle crystals suitable for single crystal XRD analysis.

Isolated yield: 63 mg, 58%

^{1}H NMR (400 MHz, 298 K, C_6D_6): δ = 7.13 (d, $^{3}J_{HH}$ = 9.8 Hz, 6H, *Ph*Br), 6.68 (d, $^{3}J_{HH}$ = 8.7 Hz, 6H, *Ph*Br), 0.03 (s, 27H, *Me*$_3$Si) ppm.

^{13}C{^{1}H} NMR (101 MHz, 298 K, C_6D_6): δ = 149.1 (s, *Ph*), 132.6 (s, *Ph*Br), 131.0 (s, *Ph*Br), 120.0 (s, *Ph*Br), 0.8 (d $^{3}J_{CP}$ = 7.8 Hz, *Me*$_3$Si) ppm.

^{31}P NMR (162 MHz, 298 K, C_6D_6): δ = 118.7 - 114.3 (m, 3P, *bridging*), –196.8 - –204.3 (q, $^{1}J_{PP}$ = 405.1 Hz, 1P, *apical*), –215.4 - –220.2 (m, 3P, *basal*) ppm.

^{29}Si DEPT90 NMR (79 MHz, 298 K, C_6D_6): δ = 13.6 (d, $^{2}J_{SiP}$ = 29.8 Hz, *Si*Me$_3$) ppm.

Mass spectrometry (ESI pos/neg): For $C_{27}H_{40}Br_3N_3P_7Si_3$ ([M+H]$^+$) Calcd.: 947.8199; found: 947.8213.

Elemental analysis: For $C_{27}H_{42}Br_3N_3P_7Si_3$: Calcd.: C 34.16, H 4.46, N 4.43; found: C 34.53, H 4.11, N 4.31.

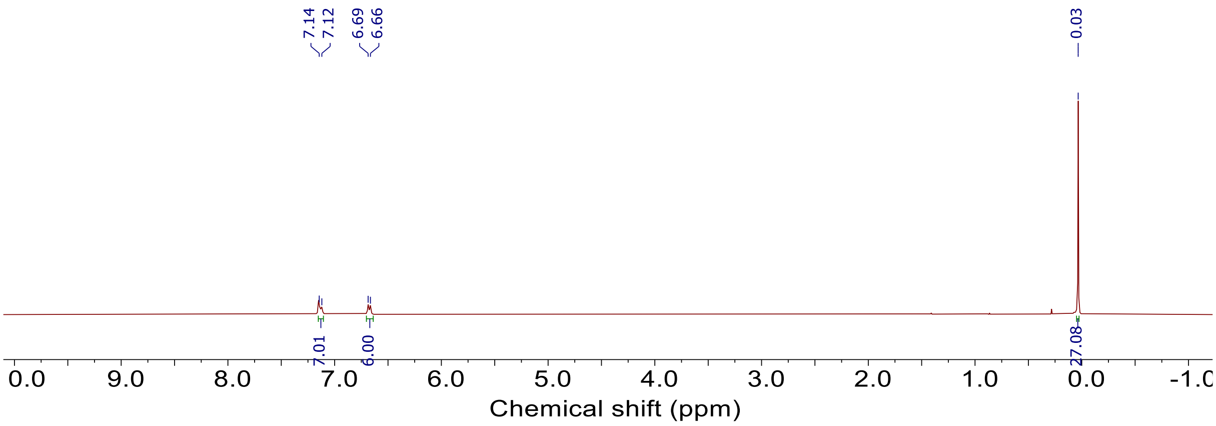

Figure S30. ^{1}H NMR spectrum (C_6D_6) of **8.**

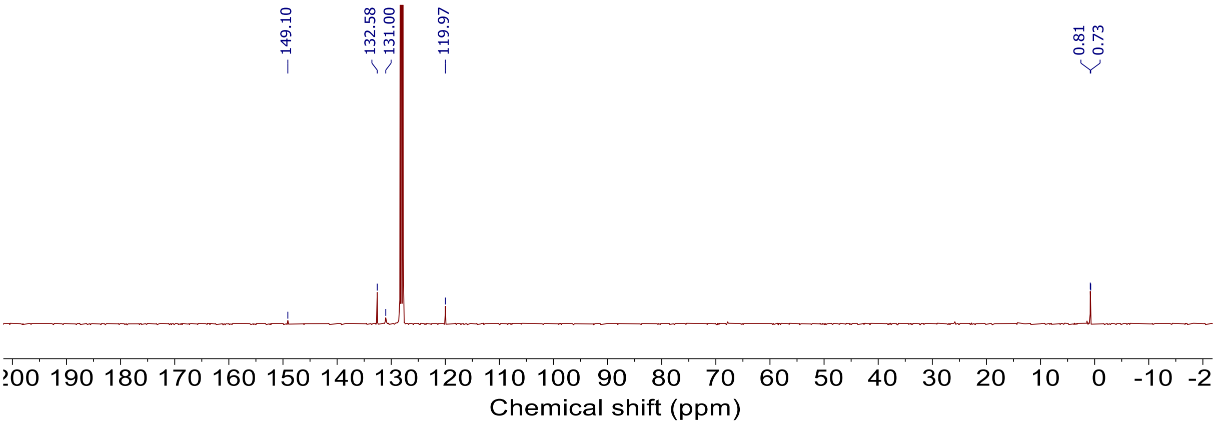

Figure S31. ^{13}C{^{1}H} NMR spectrum (C_6D_6) of **8**.

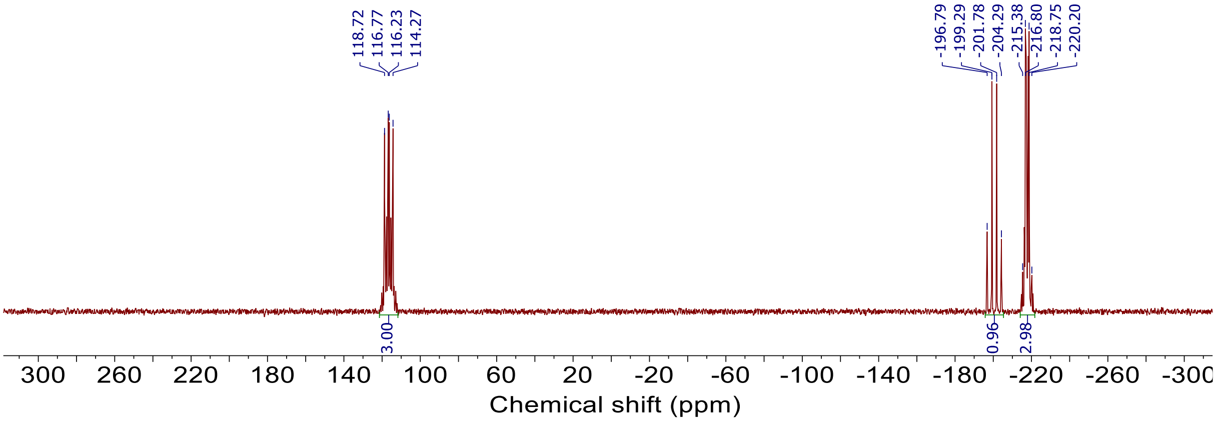

Figure S32. ^{31}P NMR spectrum (C_6D_6) of **8**.

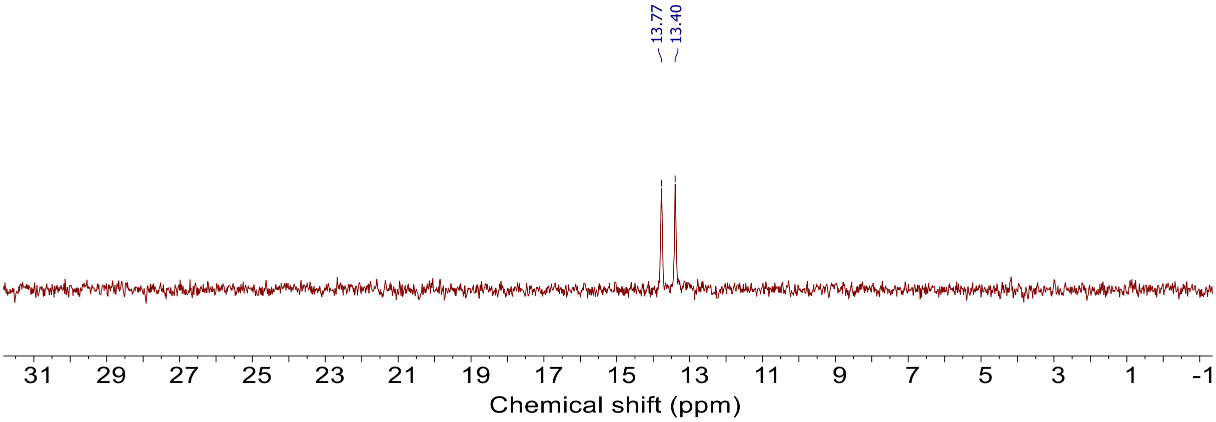

Figure S33. ^{29}Si DEPT90 NMR spectrum (C_6D_6) of **8**.

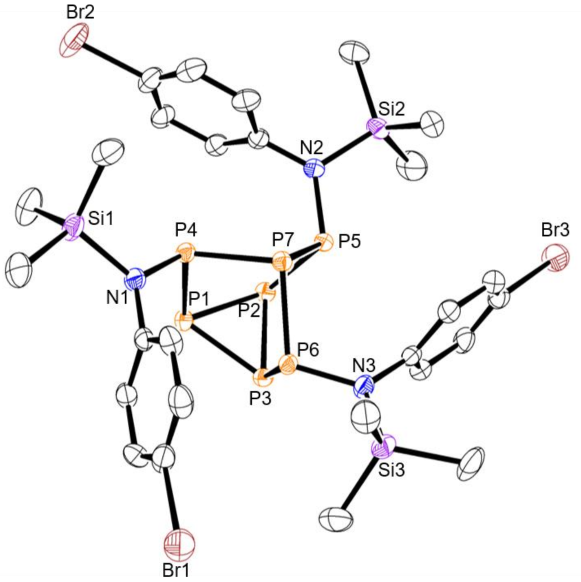

Figure S34. Molecular structure of **8**. Anisotropic displacement ellipsoids captured at 50% probability. Hydrogen atoms have been omitted for clarity. Phosphorus: orange; Nitrogen: blue; Silicon: purple; Bromine: brown Carbon: black.

3. Quantification of *mono-*, *bis-* and *tris*-inserted product distributions as azide stiochiometry is tuned

3.1. General procedure

To probe whether the distribution of *mono-, bis-* and *tris-* inserted products could be tuned with azide stoichiometry, reactions were conducted where different molar equivalents of azide were employed. A vial was loaded with triphenylphosphine oxide, as an internal standard, (5 mg, 0.018 mmol) and the cluster, either $(Me_3Si)_3P_7$ (**1**) or $(Me_3Ge)_3P_7$ (**2**). The contents of the vial were dissolved in THF (0.2 mL). In a separate vial either benzyl azide, phenyl azide or 4-bromophenyl azide was added depending on the reaction, and then dissolved in THF (0.2 mL) and cooled in the freezer to –35 °C. The contents of the two vials were mixed and left to react, until no more gas production was observed. At this point, the reaction mixture was loaded into a J Young NMR tube and investigated by ^{31}P NMR spectroscopy. Use of an internal standard is necessary as common cluster decomposition products include the generation of insoluble polyphosphides. The internal standard was also independently reacted with clusters **1** and **2** as well as inserted product **3**. No reaction was observed in each case, confirming the internal standard to be inert.

3.2. Product distributions from the reactions of clusters 1 and 2 with benzyl azide

Table S1. Product distribution of the reaction of clusters **1** and **2** with 1, 2 and 3 equivalents of BnN_3.

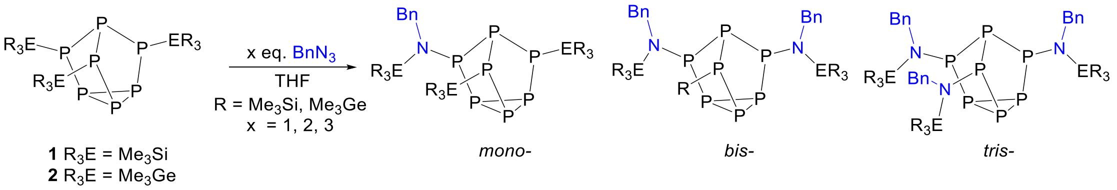

	x eq.	Starting material	*Mono-*	*Bis-*	*Tris-*	*decomposition*
**$(Me_3Si)_3P_7$ (1)**	1	43%	23%	4%	0%	30%
	2	0%	13%	17%	2%	68%
	3	0%	0%	3%	22%	74%
**$(Me_3Ge)_3P_7$ (2)**	1	31%	42%	9%	0%	18%

2	0%	0%	51%	11%	38%
3	0%	0%	0%	40%	60%

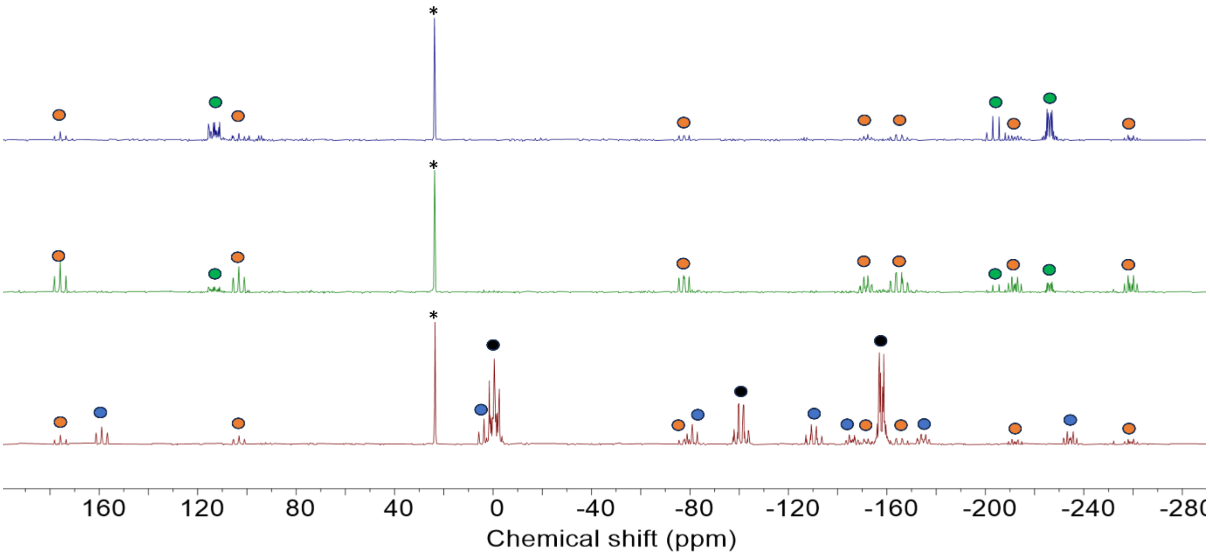

Figure S35. Stacked ^{31}P NMR spectra (THF) of **1** with (bottom to top) 1, 2 and 3 eq. of benzyl azide. **1**: black circles; **mono-**: blue circles; **bis-**: orange circles; **tris-**: green circles; triphenylphosphine oxide: asterisk.

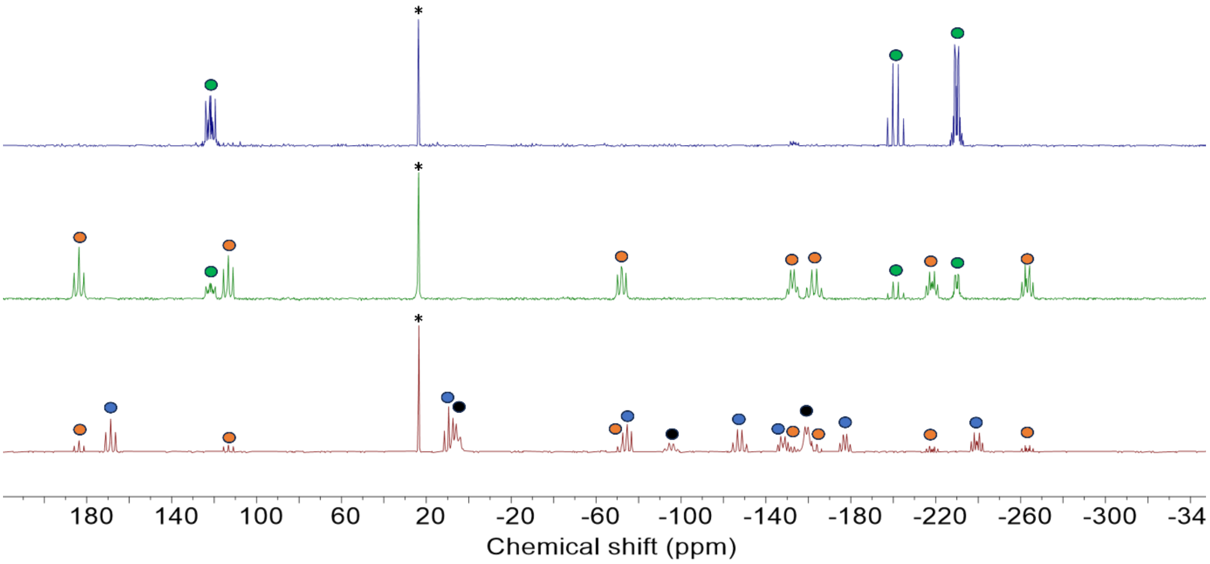

Figure S36. Stacked ^{31}P NMR spectra (THF) of **3** with (bottom to top) 1, 2 and 3 eq. benzyl azide. **2**: black circles; ***mono-***: blue circles; ***bis-***: orange circles; ***tris-***: green circles; triphenylphosphine: asterisk.

3.3. Product distributions from the reactions of clusters 1 and 2 with phenyl azide

Table S2. Product distribution of the reaction of clusters **1** and **2** with different equivalents of PhN_3.

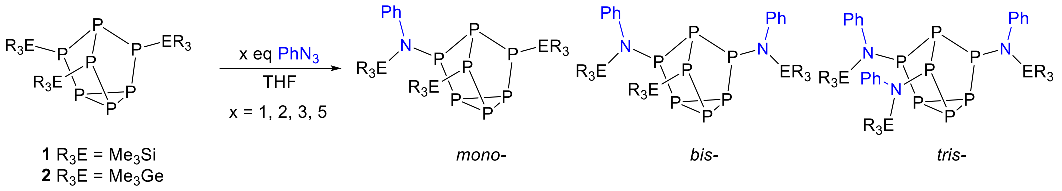

	x eq.	Starting material	*Mono-*	*Bis-*	*Tris-*	*Other*
**$(Me_3Si)_3P_7$ (1)**	1	2%	65%	30%	2%	1%
	2	0%	0%	73%	23%	4%
	3	0%	0%	0%	96%	4%
**$(Me_3Ge)_3P_7$ (2)**	1	18%	79%	3%	0%	0%
	2	0%	68%	32%	0%	0%
	3	0%	30%	68%	0%	2%
	5	0%	0%	73%	11%	17%

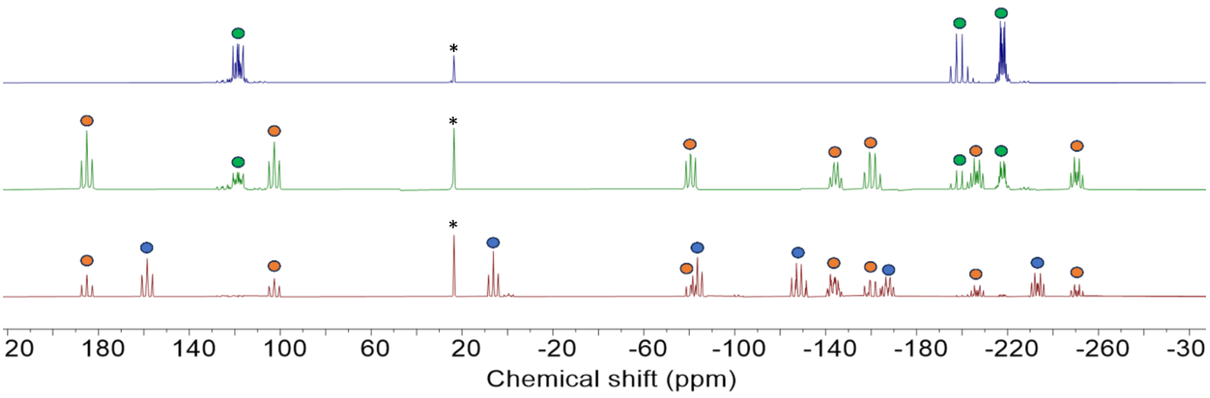

Figure S37. Stacked ^{31}P NMR spectra (THF) for the reaction of **1** with (bottom to top) 1, 2 and 3 eq. of phenyl azide. ***mono-***: blue circles; ***bis-***: orange circles; ***tris-***: green circles; triphenylphosphine oxide: asterisk.

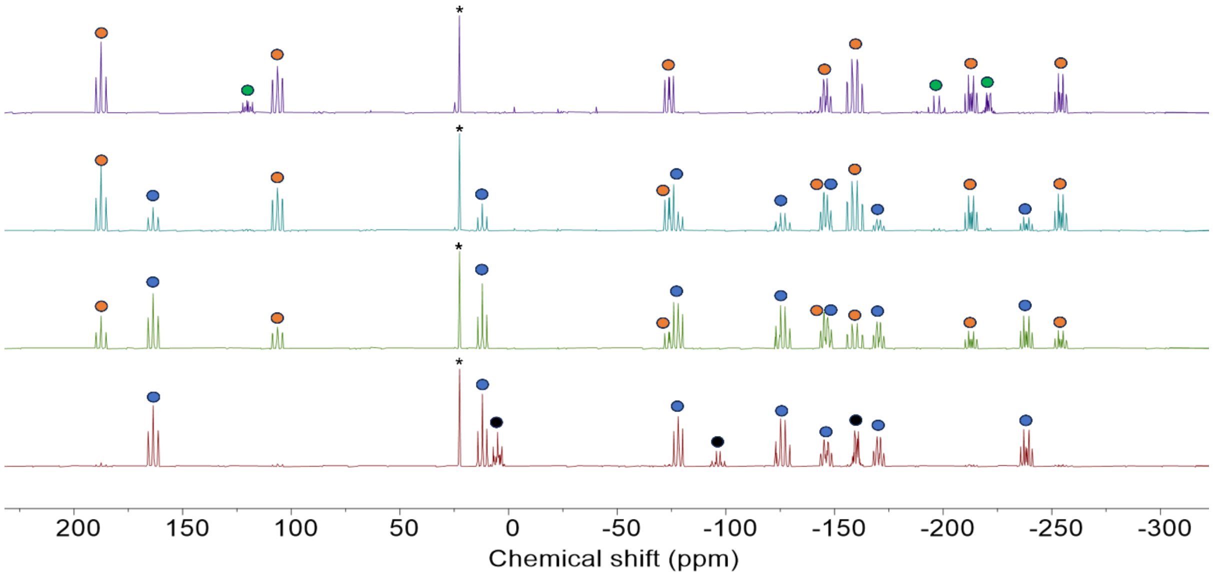

Figure S38. Stacked ^{31}P NMR spectra (THF) of **2** with (bottom to top) 1, 2, 3 and 5 eq. of phenyl azide. **2**: black circles; ***mono-***: blue circles; ***bis-***: orange circles; ***tris-***: green circles; triphenylphosphine oxide: asterisk.

3.4. Product distributions from the reactions of clusters 1 and 2 with 4-bromophenyl azide

Table S3. Product distribution of the reaction of clusters **1** and **2** with different equivalents of 4-BrPhN$_3$.

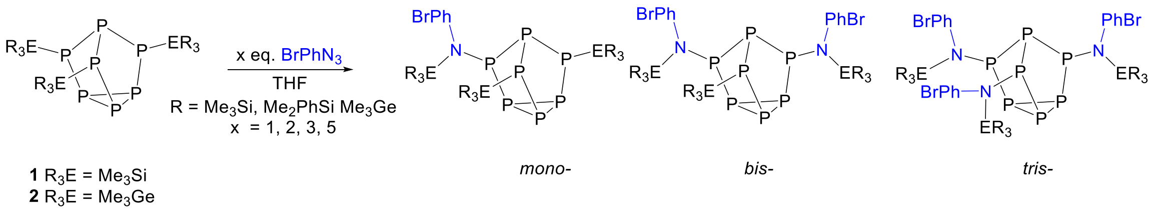

	x eq.	Starting material	*Mono-*	*Bis-*	*Tris-*	*Other*
**($Me_3Si)_3P_7$, (1)**	1	40%	36%	16%	3%	5%
	2	0%	10%	46%	33%	11%

	3	0%	0%	0%	88%	12%
	5	0%	0%	0%	99%	1%
**$(Me_3Ge)_3P_7$, (3)**	1	30%	59%	10%	0%	1%
	2	4%	45%	45%	2%	4%
	3	0%	0%	70%	23%	7%
	5	0%	0%	51%	46%	3%

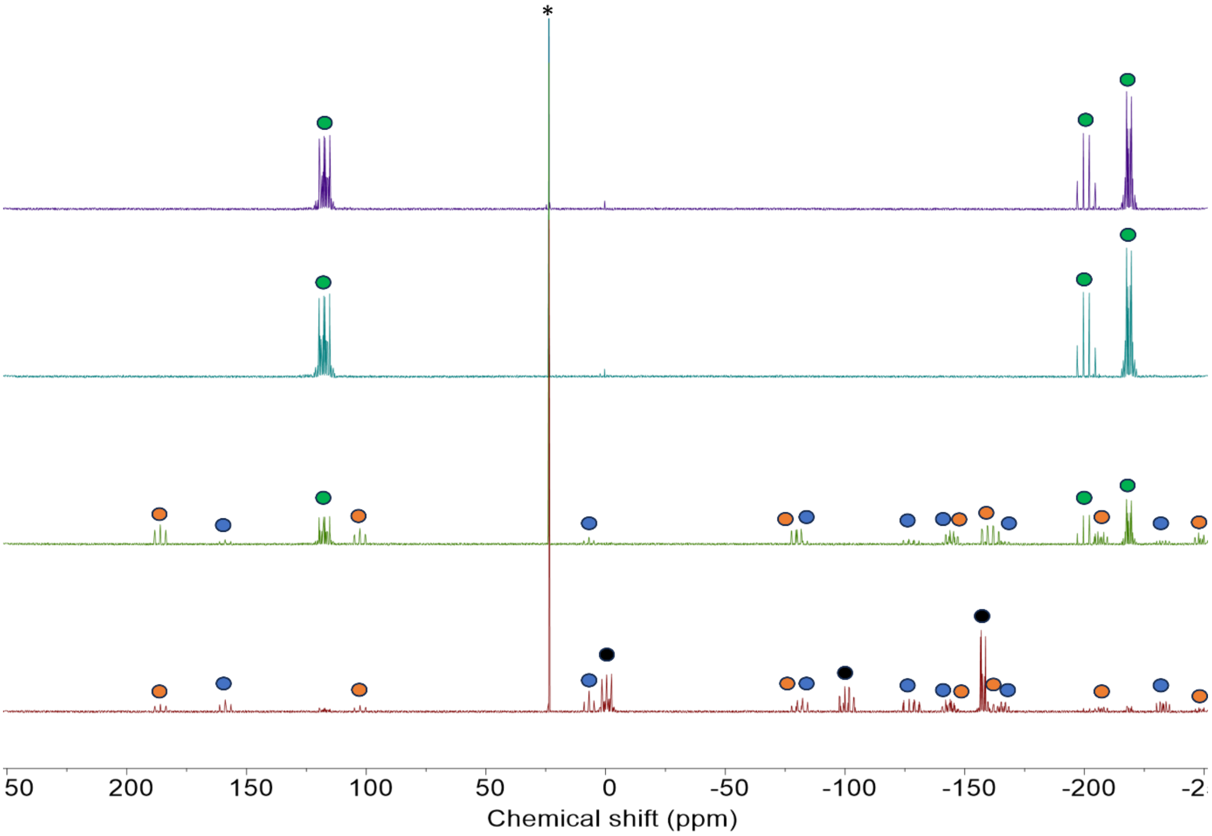

Figure S39. Stacked ^{31}P NMR spectra (THF) of **1** with (bottom to top) 1, 2 and 3 eq. of 4-bromophenyl azide. **1**: black circles; ***mono-***: blue circles; ***bis-***: orange circles; ***tris-***: green circles; triphenylphosphine oxide: asterisk.

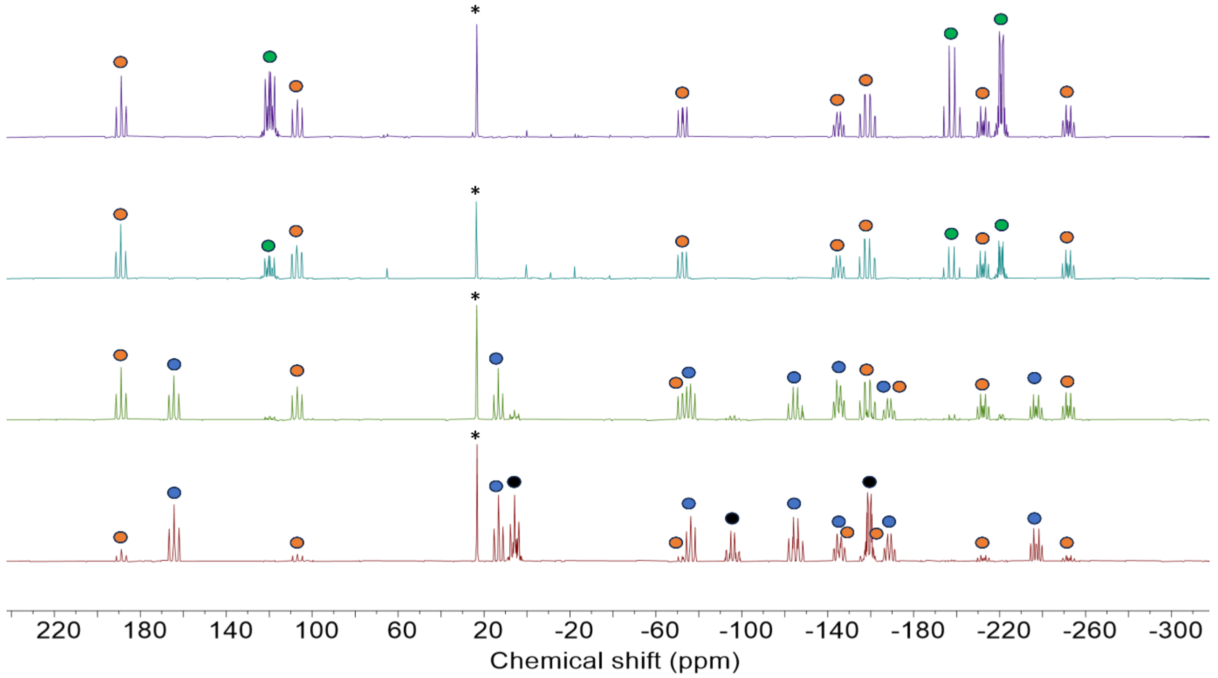

Figure S40. Stacked ^{31}P NMR spectra (THF) of **2** with (bottom to top) 1, 2, 3 and 5 eq. of 4-bromophenyl azide. **2**: black circles; ***mono-***: blue circles; ***bis-***: orange circles; ***tris-***: green circles; triphenylphosphine oxide: asterisk.

4. Atoms in Molecules calculations

Table S4. Atoms In Molecules (AIM) charges from basin integrations of individual P atoms of **1**, **2**, **3**, **4**, and **6**.

	**1**	**2**	**3**	**4**	**6**
Total electron count for $[P_7]$ cluster	106.8	106.0	103.1	103.1	105.1
P_{apical}	-0.024	-0.032	-0.091	-0.113	-0.070
$P_{bridging}$ [a]	-0.548	-0.287	0.720	0.736	0.726
$P_{bridging}$	-0.548	-0.287	0.719	0.736	-0.281
$P_{bridging}$	-0.547	-0.287	0.719	0.735	-0.298
P_{basal}	-0.042	-0.040	-0.061	-0.070	-0.044
P_{basal}	-0.039	-0.045	-0.061	-0.070	-0.054
P_{basal}	-0.041	-0.043	-0.060	-0.070	-0.030

[a] For **6**, this bridging P atom of the cluster is bonded to a N atom.

Table S5. Natural Population Analysis (NPA) charges for individual P atoms of **1**, **2**, **3**, **4**, and **6**.

	**1**	**2**	**3**	**4**	**6**
P_{apical}	0.034	0.024	-0.068	-0.108	0.003
$P_{bridging}$ [a]	-0.228	-0.202	0.457	0.472	0.488
$P_{bridging}$	-0.227	-0.202	0.457	0.461	-0.213
$P_{bridging}$	-0.227	-0.202	0.457	0.462	-0.344
P_{basal} [b]	-0.015	-0.018	-0.040	-0.049	-0.105
P_{basal}	-0.015	-0.017	-0.040	-0.048	0.017
P_{basal}	-0.015	-0.017	-0.040	-0.050	0.029

[a] For **6**, this bridging P atom is bonded to a N atom.

[b] For **6**, this basal P atom is attached to the P atom that is bonded to a N atom.

5. Optimized structures at the PBE0/6-311G(d,p) level of theory.

BnN_3

Charge = 0 Multiplicity = 1

C	-2.1353150000	3.8210910000	-0.4991020000
C	1.3243350000	4.1675870000	-1.9860370000
H	2.2429530000	4.6992210000	-1.7605850000
C	0.2269450000	4.3014640000	-1.1478870000
H	0.2954900000	4.9382130000	-0.2695310000
C	1.2477260000	3.3499480000	-3.1087640000
H	2.1056240000	3.2415340000	-3.7639430000
C	-1.0334150000	2.8077120000	-2.5440610000
H	-1.9546270000	2.2758950000	-2.7522490000
C	-0.9619400000	3.6250640000	-1.4214500000
C	0.0681300000	2.6714140000	-3.3825580000
H	0.0009770000	2.0287540000	-4.2543770000
N	-3.2100030000	2.8784130000	-0.8181980000
N	-4.2621590000	3.0478000000	-0.2210710000
N	-5.2790960000	3.1068890000	0.2704170000
H	-1.8105530000	3.6787100000	0.5402760000
H	-2.5020710000	4.8532910000	-0.5878800000

PhN$_3$

Charge = 0 Multiplicity = 1

N	-1.9754750000	3.7936960000	-0.2694960000
C	1.1945150000	4.1730100000	-2.0660070000
H	2.1071450000	4.7494950000	-1.9582480000
C	0.1812810000	4.3208920000	-1.1318090000
H	0.2799040000	5.0011060000	-0.2936690000
C	1.0469380000	3.2941920000	-3.1341660000
H	1.8415550000	3.1809590000	-3.8631420000
C	-1.1494350000	2.7006000000	-2.3317250000
H	-2.0630140000	2.1239220000	-2.4388470000
C	-0.9945840000	3.5839310000	-1.2629390000
C	-0.1272720000	2.5617090000	-3.2603790000
H	-0.2532590000	1.8733510000	-4.0894380000
N	-3.0243780000	3.1649030000	-0.3482350000
N	-4.0279200000	2.6482340000	-0.3099010000

4-BrPhN$_3$

Charge = 0 Multiplicity = 1

N	-2.0199110000	3.7971800000	-0.2325390000
C	1.1546780000	4.1848500000	-2.0209560000
H	2.0686450000	4.7573240000	-1.9196940000
C	0.1362650000	4.3255150000	-1.0928000000
H	0.2388520000	5.0068440000	-0.2562680000
C	0.9961870000	3.3040900000	-3.0838060000
Br	2.3861720000	3.1092410000	-4.3540560000
C	-1.1886010000	2.7072860000	-2.2941050000
H	-2.0989120000	2.1271950000	-2.4084210000
C	-1.0399630000	3.5888780000	-1.2231500000
C	-0.1700230000	2.5648200000	-3.2247860000
H	-0.2849070000	1.8805080000	-4.0565780000
N	-3.0688680000	3.1666380000	-0.3135260000
N	-4.0716160000	2.6496300000	-0.2763150000

$(Me_3Si)_3P_7$

Charge = 0 Multiplicity = 1

P	0.8702640000	0.1083260000	-0.7812890000
P	2.5911350000	1.0391400000	0.2229430000
P	2.0575540000	0.8741820000	2.3659190000

Si	2.4720250000	3.2890010000	-0.1958170000
C	2.0969860000	3.4583540000	-2.0270600000
H	2.8003860000	2.8855700000	-2.6377030000
H	2.1727020000	4.5103170000	-2.3231050000
H	1.0858160000	3.1139080000	-2.2596660000
C	4.2031810000	3.9102530000	0.1839500000
H	4.4670310000	3.7440570000	1.2324720000
H	4.2667510000	4.9862020000	-0.0110220000
H	4.9500580000	3.4074910000	-0.4364420000
C	1.2133020000	4.2208550000	0.8333230000
H	0.2007630000	3.8605670000	0.6377390000
H	1.2604780000	5.2857920000	0.5785610000
H	1.4142440000	4.1222320000	1.9038880000
P	-0.7216220000	1.1181400000	0.3513000000
P	-0.1654120000	0.7049060000	2.4546090000
Si	-2.6397850000	-0.0996460000	0.0542840000
C	-2.7435390000	-0.4742710000	-1.7820360000
H	-2.6425250000	0.4317970000	-2.3858780000
H	-3.7155290000	-0.9247850000	-2.0106350000
H	-1.9653260000	-1.1777590000	-2.0902140000
C	-4.0048110000	1.0840390000	0.5661070000
H	-3.9095500000	1.3738980000	1.6165990000
H	-4.9829230000	0.6080270000	0.4373220000
H	-3.9874510000	1.9944830000	-0.0390270000
C	-2.7438570000	-1.6760720000	1.0623010000
H	-1.9491230000	-2.3726790000	0.7868170000
H	-3.7108690000	-2.1570860000	0.8758620000
H	-2.6703700000	-1.4754480000	2.1348710000
P	0.8688630000	-1.7933090000	0.3238900000
P	1.0926890000	-1.1377780000	2.4271530000
Si	2.8625210000	-2.8304230000	-0.1287750000
C	3.1321900000	-2.6703410000	-1.9795540000
H	2.2645060000	-3.0176040000	-2.5471030000
H	3.9947980000	-3.2759110000	-2.2784360000
H	3.3328860000	-1.6341100000	-2.2645490000
C	2.5544150000	-4.6221520000	0.3414380000
H	2.3198560000	-4.7221110000	1.4052280000
H	3.4477460000	-5.2226050000	0.1380010000
H	1.7232190000	-5.0448100000	-0.2295270000
C	4.3346830000	-2.1622580000	0.8194030000

H	4.5191570000	-1.1141200000	0.5730300000
H	5.2244750000	-2.7446410000	0.5542580000
H	4.1900050000	-2.2436190000	1.9005670000

$(Me_3Ge)_3P_7$

Charge = 0 Multiplicity = 1

Ge	1.0601000000	3.4303890000	0.1529390000
Ge	2.5525550000	-2.5715630000	-0.2024480000
P	-1.1724370000	-1.4854750000	-0.0070380000
P	1.9315830000	-0.3602020000	0.3317310000
P	-0.6126600000	1.7723580000	0.2976580000
P	0.1346250000	0.0610880000	-0.8618110000
P	-1.0373280000	-1.0832980000	2.1681360000
P	1.1151060000	-0.5441270000	2.3840470000
P	-0.4394730000	1.0532720000	2.3864440000
C	2.5889850000	3.1493190000	1.3371670000
H	3.2729090000	3.9970950000	1.2373230000
H	2.2707690000	3.0801570000	2.3793980000
C	0.1275810000	5.0861080000	0.6220300000
H	-0.2650410000	5.0356610000	1.6397540000
H	0.8217630000	5.9285590000	0.5597610000
C	1.5151130000	-3.9489430000	0.7154550000
H	1.9352320000	-4.9285790000	0.4701920000
H	1.5548410000	-3.8146770000	1.7983690000
C	1.6295620000	3.4828590000	-1.7171130000
H	2.1619760000	2.5682130000	-1.9842520000
H	0.7731410000	3.5988450000	-2.3840690000
C	2.3801280000	-2.7471210000	-2.1429310000
H	2.7967760000	-3.7089750000	-2.4542920000
H	1.3311570000	-2.7086570000	-2.4424170000
H	4.8308270000	-3.6625380000	0.0777940000
H	5.0214790000	-1.9221810000	-0.2169410000
C	-3.3363330000	-0.2188310000	-2.4057790000
H	-2.8870700000	-0.9720090000	-3.0559350000
H	-3.5477090000	1.4794430000	0.5086520000
C	4.4362990000	-2.6717310000	0.3191740000
Ge	-3.3737940000	-0.8277250000	-0.5472160000
C	-4.1138740000	0.5538810000	0.6188360000
H	-4.0877820000	0.2417600000	1.6648420000
C	-4.4129580000	-2.4795560000	-0.4003760000

H	-4.3806440000	-2.8711060000	0.6184660000
H	-5.4556780000	-2.2813390000	-0.6627350000
H	-2.7667890000	0.7079650000	-2.4951170000
H	0.4747000000	-3.9135780000	0.3908170000
H	2.9218710000	-1.9511340000	-2.6574480000
H	4.5527930000	-2.5047870000	1.3919550000
H	-4.0195250000	-3.2385730000	-1.0791530000
H	-4.3597530000	-0.0333920000	-2.7433930000
H	-5.1547260000	0.7339200000	0.3347850000
H	-0.7021530000	5.2654050000	-0.0643230000
H	2.3012290000	4.3334940000	-1.8627670000
H	3.1126260000	2.2323040000	1.0638300000

$(Me_3SiNBn)_3P_7$

Charge = 0 Multiplicity = 1

Si	4.2111020000	-1.9457910000	0.1625360000
Si	-3.7880970000	-2.6746600000	0.1621710000
Si	-0.4185520000	4.6184730000	0.1551660000
P	-0.0002860000	0.0012550000	-0.0799340000
P	1.8754940000	-0.4236450000	1.0235650000
P	-1.3036850000	-1.4112570000	1.0259850000
P	-0.5692420000	1.8367280000	1.0262200000
P	1.1593360000	-0.5362320000	3.1816560000
P	-1.0400080000	-0.7344460000	3.1833470000
P	-0.1120040000	1.2691480000	3.1831860000
N	2.4784270000	-1.9522830000	0.6104970000
N	-2.9303010000	-1.1706540000	0.6157880000
N	0.4526930000	3.1241590000	0.6142310000
C	4.4659180000	-1.0146530000	-1.4417500000
H	3.9653370000	-1.5145920000	-2.2761440000
H	5.5335950000	-0.9535810000	-1.6789980000
H	4.0824620000	0.0084330000	-1.3767630000
C	5.2021880000	-1.1251300000	1.5274830000
H	4.9347320000	-0.0701910000	1.6341940000
H	6.2725730000	-1.1775690000	1.3007130000
H	5.0367190000	-1.6163520000	2.4911540000
C	4.7480650000	-3.7320660000	-0.0281500000
H	4.7030260000	-4.2820780000	0.9164830000
H	5.7887120000	-3.7508590000	-0.3684820000
H	4.1458250000	-4.2659490000	-0.7684830000

C	1.7181600000	-3.1952040000	0.6998930000
H	0.8360780000	-3.0222240000	1.3277950000
H	2.3164120000	-3.9533240000	1.2189430000
C	1.2498340000	-3.7747490000	-0.6166030000
C	1.2072540000	-3.0257870000	-1.7889400000
H	1.5424000000	-1.9945850000	-1.7740830000
C	0.7434390000	-3.5897130000	-2.9738050000
H	0.7176300000	-2.9903590000	-3.8783220000
C	0.3151290000	-4.9101140000	-3.0019080000
H	-0.0445010000	-5.3499110000	-3.9262120000
C	0.3533930000	-5.6662800000	-1.8343960000
H	0.0251910000	-6.7007470000	-1.8450500000
C	0.8186170000	-5.1019830000	-0.6547190000
H	0.8519480000	-5.7006220000	0.2521230000
C	-3.1006550000	-3.3588950000	-1.4393580000
H	-3.2848900000	-2.6760890000	-2.2739930000
H	-3.5751910000	-4.3167790000	-1.6785860000
H	-2.0220520000	-3.5321830000	-1.3718110000
C	-3.5816930000	-3.9419450000	1.5296850000
H	-2.5353910000	-4.2383020000	1.6439130000
H	-4.1616740000	-4.8426730000	1.3011710000
H	-3.9301530000	-3.5504810000	2.4902550000
C	-5.6022620000	-2.2467160000	-0.0393850000
H	-6.0630750000	-1.9345470000	0.9024720000
H	-6.1360390000	-3.1381910000	-0.3852180000
H	-5.7585580000	-1.4571340000	-0.7796100000
C	-3.6280770000	0.1081080000	0.7085120000
H	-3.0363750000	0.7853930000	1.3357120000
H	-4.5822230000	-0.0326620000	1.2300920000
C	-3.9005480000	0.8047630000	-0.6062000000
C	-3.2312200000	0.4731220000	-1.7805710000
H	-2.4996660000	-0.3272630000	-1.7687720000
C	-3.4952000000	1.1564150000	-2.9640790000
H	-2.9638990000	0.8831570000	-3.8702080000
C	-4.4300600000	2.1826500000	-2.9884530000
H	-4.6366680000	2.7138010000	-3.9116230000
C	-5.1024150000	2.5227340000	-1.8186730000
H	-5.8381400000	3.3205860000	-1.8262430000
C	-4.8396100000	1.8372860000	-0.6405520000
H	-5.3737230000	2.1040410000	0.2679300000

C	-1.3471820000	4.3664010000	-1.4512020000
H	-0.6612900000	4.1834740000	-2.2835410000
H	-1.9369790000	5.2574710000	-1.6921350000
H	-2.0385980000	3.5203120000	-1.3857570000
C	-1.6297620000	5.0730960000	1.5135040000
H	-2.4121180000	4.3164220000	1.6193960000
H	-2.1161160000	6.0266540000	1.2809140000
H	-1.1254910000	5.1775560000	2.4789400000
C	0.8603210000	5.9761340000	-0.0374760000
H	1.3563160000	6.2169320000	0.9074460000
H	0.3567170000	6.8847580000	-0.3838760000
H	1.6260090000	5.7183540000	-0.7744600000
C	1.9093210000	3.0868920000	0.7045960000
H	2.1995600000	2.2353700000	1.3315620000
H	2.2669590000	3.9834970000	1.2246480000
C	2.6468220000	2.9715010000	-0.6109830000
C	2.0222050000	2.5563660000	-1.7836200000
H	0.9622510000	2.3278750000	-1.7701120000
C	2.7445110000	2.4362430000	-2.9671670000
H	2.2403060000	2.1114960000	-3.8717770000
C	4.1018070000	2.7272510000	-2.9935150000
H	4.6640570000	2.6351150000	-3.9167890000
C	4.7350240000	3.1415890000	-1.8256510000
H	5.7946880000	3.3759730000	-1.8349820000
C	4.0115980000	3.2634420000	-0.6474430000
H	4.5115890000	3.5939040000	0.2595330000

$(Me_3GeNBn)_3P_7$

Charge = 0 Multiplicity = 1

Ge	-3.8427710000	2.7447480000	-0.1184850000
Ge	-0.4492230000	-4.7031390000	-0.1234890000
Ge	4.3103440000	1.9667840000	-0.1304080000
P	0.0028380000	0.0024810000	0.0652670000
P	-1.3103500000	1.4134560000	-1.0342570000
P	-0.5579900000	-1.8415810000	-1.0334490000
P	1.8850030000	0.4371220000	-1.0255720000
P	-1.0298470000	0.7380710000	-3.1956980000
P	-0.1027730000	-1.2650470000	-3.1938390000
P	1.1681080000	0.5394660000	-3.1894540000
N	-2.9239910000	1.1619300000	-0.6222480000

N	0.4639120000	-3.1129460000	-0.6134570000
N	2.4721410000	1.9586310000	-0.6037120000
C	-3.1168660000	3.3857350000	1.5727310000
H	-3.3326560000	2.6790190000	2.3769210000
H	-3.5639760000	4.3507870000	1.8275580000
H	-2.0345670000	3.5178370000	1.4974200000
C	-3.5676730000	4.0625010000	-1.5317800000
H	-2.5108700000	4.3278150000	-1.6020310000
H	-4.1401320000	4.9684130000	-1.3131000000
H	-3.8946580000	3.6665190000	-2.4959500000
C	-5.7285680000	2.2674180000	0.0380250000
H	-6.1467190000	1.9751960000	-0.9279770000
H	-6.2805060000	3.1404300000	0.3970190000
H	-5.8714440000	1.4516540000	0.7496220000
C	-3.6346970000	-0.1048060000	-0.7141260000
H	-3.0320340000	-0.8024260000	-1.3090230000
H	-4.5724360000	0.0339480000	-1.2672450000
C	-3.9532410000	-0.7738830000	0.6050820000
C	-3.2632620000	-0.4724700000	1.7763190000
H	-2.4876700000	0.2854310000	1.7558300000
C	-3.5610120000	-1.1331210000	2.9643500000
H	-3.0129380000	-0.8844810000	3.8676590000
C	-4.5508640000	-2.1066520000	2.9972820000
H	-4.7835580000	-2.6201540000	3.9242780000
C	-5.2446440000	-2.4156230000	1.8316540000
H	-6.0238840000	-3.1710200000	1.8461990000
C	-4.9485020000	-1.7517760000	0.6488040000
H	-5.4999760000	-1.9928840000	-0.2565240000
C	-1.4114100000	-4.3902880000	1.5421980000
H	-0.7126220000	-4.2164470000	2.3633720000
H	-2.0257640000	-5.2616170000	1.7860970000
H	-2.0687310000	-3.5223850000	1.4464660000
C	-1.6882850000	-5.1417970000	-1.5662810000
H	-2.4456820000	-4.3616600000	-1.6664600000
H	-2.1910740000	-6.0888940000	-1.3508950000
H	-1.1551800000	-5.2367630000	-2.5150250000
C	0.9123650000	-6.0869050000	0.0767950000
H	1.3987160000	-6.3083080000	-0.8760060000
H	0.4301670000	-7.0015510000	0.4325620000
H	1.6712920000	-5.7908650000	0.8038740000

C	1.9171310000	-3.0921420000	-0.6886990000
H	2.2253620000	-2.2224460000	-1.2823030000
H	2.2744180000	-3.9744570000	-1.2350160000
C	2.6395450000	-3.0275810000	0.6393390000
C	2.0159990000	-2.5825300000	1.8019180000
H	0.9704580000	-2.2964940000	1.7669630000
C	2.7213470000	-2.5032750000	2.9988390000
H	2.2186360000	-2.1537830000	3.8951110000
C	4.0608320000	-2.8662240000	3.0495000000
H	4.6097870000	-2.8059600000	3.9834080000
C	4.6926210000	-3.3116670000	1.8927450000
H	5.7377880000	-3.6029620000	1.9213710000
C	3.9854870000	-3.3929440000	0.7007470000
H	4.4834510000	-3.7488400000	-0.1977120000
C	4.5386540000	0.9507880000	1.5167560000
H	4.0516170000	1.4590590000	2.3518400000
H	5.6028760000	0.8462560000	1.7459760000
H	4.1095250000	-0.0488040000	1.4120260000
C	5.2999710000	1.1450660000	-1.5982690000
H	5.0027000000	0.1011810000	-1.7181720000
H	6.3734910000	1.1795530000	-1.3922440000
H	5.1063070000	1.6738300000	-2.5343660000
C	4.8169850000	3.8375310000	0.0994580000
H	4.7606800000	4.3851060000	-0.8441090000
H	5.8502990000	3.8782720000	0.4547680000
H	4.1785630000	4.3300410000	0.8358200000
C	1.7250050000	3.2047490000	-0.6820440000
H	0.8247980000	3.0379490000	-1.2865940000
H	2.3147820000	3.9590140000	-1.2185720000
C	1.2913370000	3.7921140000	0.6434650000
C	1.2449820000	3.0361190000	1.8114950000
H	1.5512590000	1.9961750000	1.7825510000
C	0.8102070000	3.6030180000	3.0059490000
H	0.7803720000	2.9981690000	3.9067270000
C	0.4142220000	4.9333690000	3.0483300000
H	0.0770520000	5.3752110000	3.9801290000
C	0.4562010000	5.6965340000	1.8855670000
H	0.1535110000	6.7386160000	1.9074930000
C	0.8932500000	5.1292570000	0.6962400000
H	0.9300090000	5.7332480000	-0.2069400000

$(Me_3SiNPh)_3P_7$

Charge = 0 Multiplicity = 1

Si	-4.2356030000	1.2649850000	-0.9153400000
Si	0.5143920000	-4.0588420000	-0.2388680000
Si	3.0726040000	3.0158060000	-0.9109700000
P	-1.7999700000	-0.7847370000	-0.5458060000
P	1.5272420000	-1.0159440000	-0.5322090000
P	0.7953970000	-0.8300560000	-2.6724170000
P	-1.3128060000	-0.1635350000	-2.6583380000
P	-0.0711320000	-0.0097390000	0.6080930000
P	0.0983010000	1.9468300000	-0.4249220000
P	0.3184550000	1.3274150000	-2.5972480000
N	-3.2093290000	0.0805490000	-0.0581770000
N	1.6446630000	-2.6750570000	-0.1243360000
N	1.6192650000	2.6231490000	0.0477610000
C	-5.1650230000	-2.5014070000	1.8069020000
H	-5.7102660000	-3.4175100000	1.6044110000
C	-4.5471750000	-1.8233900000	0.7642330000
H	-4.5914640000	-2.2054710000	-0.2506140000
C	2.9907790000	-2.9968630000	1.9063840000
H	2.0970370000	-2.8208070000	2.4961050000
C	-5.0862920000	-2.0088510000	3.1049300000
H	-5.5699900000	-2.5387910000	3.9186240000
C	3.7813500000	1.5306410000	-1.8017230000
H	3.9290730000	0.6869870000	-1.1237310000
H	4.7566890000	1.8247050000	-2.2070190000
H	3.1718160000	1.1912950000	-2.6424420000
C	0.7814480000	4.6043670000	1.2577570000
H	0.3897440000	4.9885220000	0.3213980000
C	4.2075460000	-3.2671100000	2.5221460000
H	4.2628640000	-3.3149820000	3.6048830000
C	0.6297330000	5.3202850000	2.4380200000
H	0.1234240000	6.2800200000	2.4236310000
C	-3.7559300000	-0.1607370000	2.3158430000
H	-3.1896690000	0.7459590000	2.5000630000
C	4.0549240000	-3.1297930000	-0.2476300000
H	3.9805820000	-3.0709990000	-1.3286780000
C	1.7673120000	3.5782580000	3.6430930000
H	2.1499470000	3.1717250000	4.5735970000

C	5.2702990000	-3.3918980000	0.3699090000
H	6.1588860000	-3.5424810000	-0.2344870000
C	-5.4430360000	1.9577780000	0.3391570000
H	-6.0686170000	1.1797600000	0.7835370000
H	-6.1019260000	2.6752330000	-0.1619620000
H	-4.9286160000	2.4827200000	1.1487500000
C	-3.8458490000	-0.6420980000	1.0108000000
C	-0.3908230000	-4.0271050000	-1.8772240000
H	-1.0913350000	-3.1934310000	-1.9491440000
H	-0.9726160000	-4.9512780000	-1.9675040000
H	0.3002510000	-3.9826250000	-2.7238440000
C	-3.2528300000	2.6750900000	-1.6532670000
H	-2.5638290000	3.1157680000	-0.9290810000
H	-3.9699350000	3.4458990000	-1.9592980000
H	-2.6846220000	2.3934810000	-2.5430640000
C	1.4364370000	3.3717170000	1.2612310000
C	1.1258280000	4.8112540000	3.6330920000
H	1.0083460000	5.3721530000	4.5542530000
C	2.9055560000	-2.9334140000	0.5166720000
C	1.9174970000	2.8578650000	2.4644800000
H	2.4068830000	1.8896890000	2.4585620000
C	4.3589510000	3.6652290000	0.2867920000
H	4.0114330000	4.5442840000	0.8352910000
H	5.2524680000	3.9509920000	-0.2788630000
H	4.6520190000	2.9053650000	1.0163870000
C	5.3496960000	-3.4662130000	1.7564750000
H	6.2994270000	-3.6742940000	2.2376270000
C	-0.7047380000	-4.0661420000	1.1847880000
H	-0.1913620000	-4.0604800000	2.1509070000
H	-1.3186840000	-4.9727400000	1.1413790000
H	-1.3794260000	-3.2070360000	1.1447070000
C	-4.3787470000	-0.8393030000	3.3562210000
H	-4.3053070000	-0.4546020000	4.3681680000
C	1.5633110000	-5.6118450000	-0.1649230000
C	-5.1928280000	0.3840070000	-2.2702940000
C	2.6566160000	4.3591840000	-2.1572930000
H	3.5453740000	4.6349590000	-2.7350830000
H	2.2917670000	5.2612660000	-1.6563500000
H	1.8904930000	4.0290730000	-2.8654440000
H	2.3092680000	-5.6327140000	-0.9644460000

H	0.9124350000	-6.4842460000	-0.2883160000
H	2.0890710000	-5.7158490000	0.7874240000
H	-4.5208770000	-0.0535410000	-3.0150530000
H	-5.8565790000	1.0830900000	-2.7902780000
H	-5.8122420000	-0.4173820000	-1.8559900000

$(Me_3GeNPh)(Me_3Ge)_2P_7$

Charge = 0 Multiplicity = 1

Ge	3.7290460000	-1.7984790000	-0.9974100000
Ge	-2.9716010000	-2.2175150000	0.5388940000
Ge	0.9226830000	3.5861140000	0.1455340000
P	1.4652000000	-1.7791310000	-0.3274150000
P	-1.0377920000	0.4545270000	0.3425300000
P	-0.2439940000	-0.3059430000	2.2916300000
P	1.6016910000	-1.4756700000	1.8662180000
P	0.6905170000	0.1526990000	-1.0261150000
P	1.9957630000	1.4770560000	0.1344390000
P	1.7248920000	0.7149910000	2.2266470000
N	-2.3521470000	-0.5366640000	-0.1021930000
C	-3.5014990000	0.0778970000	-2.1740330000
H	-2.8445020000	-0.6073420000	-2.6989950000
C	2.4195260000	4.8487830000	0.1689100000
H	3.0303880000	4.7115140000	1.0639860000
H	2.0358770000	5.8730400000	0.1640440000
H	3.0524520000	4.7081790000	-0.7098810000
C	-0.2206030000	3.8854060000	1.7021570000
H	-1.0937470000	3.2337350000	1.6504110000
H	-0.5513310000	4.9284640000	1.7008340000
H	0.3175450000	3.6926670000	2.6327340000
C	-4.4710200000	0.8028150000	-2.8564380000
H	-4.5796570000	0.6778030000	-3.9289080000
C	-4.1718110000	1.1259270000	-0.1145740000
H	-4.0369380000	1.2528040000	0.9548100000
C	-5.1368360000	1.8528630000	-0.7994550000
H	-5.7701440000	2.5486570000	-0.2586000000
C	4.2010710000	-3.6982100000	-1.0187890000
H	4.1013950000	-4.1339170000	-0.0221820000
H	5.2376630000	-3.8160560000	-1.3471980000
H	3.5517910000	-4.2449850000	-1.7057070000
C	3.7842300000	-1.0702980000	-2.8112660000

H	3.0800120000	-1.5931390000	-3.4617460000
H	4.7920110000	-1.1926700000	-3.2183360000
H	3.5343020000	-0.0076760000	-2.8057400000
C	-1.5093700000	-3.4336760000	0.9433320000
H	-0.7744810000	-3.4578920000	0.1371290000
H	-1.9424510000	-4.4322460000	1.0573900000
H	-1.0043040000	-3.1734070000	1.8747150000
C	4.9288170000	-0.8160730000	0.1904750000
H	4.6723470000	0.2441830000	0.1910800000
H	5.9555560000	-0.9372740000	-0.1678210000
H	4.8697550000	-1.1992150000	1.2115580000
C	-3.3481600000	0.2272340000	-0.7956730000
C	-4.0404760000	-1.8436730000	2.1313590000
H	-3.4197250000	-1.3937950000	2.9096700000
H	-4.4675120000	-2.7721400000	2.5208510000
H	-4.8587000000	-1.1612370000	1.8911040000
C	-0.0948530000	3.7866750000	-1.5115890000
H	0.5059370000	3.5135420000	-2.3814580000
H	-0.4001490000	4.8323360000	-1.6136570000
H	-0.9886250000	3.1608610000	-1.4861040000
C	-5.2922950000	1.6907750000	-2.1717080000
H	-6.0469630000	2.2584880000	-2.7055720000
C	-4.0918190000	-2.9693310000	-0.8702570000
H	-4.9125510000	-2.2975980000	-1.1267160000
H	-4.5122600000	-3.9145960000	-0.5148080000
H	-3.5056540000	-3.1689060000	-1.7698410000

$(Me_3SiNPhBr)_3P_7$

Charge = 0 Multiplicity = 1

Si	-4.2360000000	1.2650000000	-0.9150000000
Si	0.5140000000	-4.0590000000	-0.2390000000
Si	3.0730000000	3.0160000000	-0.9110000000
P	-1.8000000000	-0.7850000000	-0.5460000000
P	1.5270000000	-1.0160000000	-0.5320000000
P	0.7950000000	-0.8300000000	-2.6720000000
P	-1.3130000000	-0.1640000000	-2.6580000000
P	-0.0710000000	-0.0100000000	0.6080000000
P	0.0980000000	1.9470000000	-0.4250000000
P	0.3180000000	1.3270000000	-2.5970000000
N	-3.2090000000	0.0810000000	-0.0580000000

N	1.6450000000	-2.6750000000	-0.1240000000
N	1.6190000000	2.6230000000	0.0480000000
C	-5.1650000000	-2.5010000000	1.8070000000
H	-5.7100000000	-3.4180000000	1.6040000000
C	-4.5470000000	-1.8230000000	0.7640000000
H	-4.5910000000	-2.2050000000	-0.2510000000
C	2.9910000000	-2.9970000000	1.9060000000
H	2.0970000000	-2.8210000000	2.4960000000
C	-5.0860000000	-2.0090000000	3.1050000000
Br	-5.5700000000	-2.5390000000	3.9190000000
C	3.7810000000	1.5310000000	-1.8020000000
H	3.9290000000	0.6870000000	-1.1240000000
H	4.7570000000	1.8250000000	-2.2070000000
H	3.1720000000	1.1910000000	-2.6420000000
C	0.7810000000	4.6040000000	1.2580000000
H	0.3900000000	4.9890000000	0.3210000000
C	4.2080000000	-3.2670000000	2.5220000000
H	4.2630000000	-3.3150000000	3.6050000000
C	0.6300000000	5.3200000000	2.4380000000
H	0.1230000000	6.2800000000	2.4240000000
C	-3.7560000000	-0.1610000000	2.3160000000
H	-3.1900000000	0.7460000000	2.5000000000
C	4.0550000000	-3.1300000000	-0.2480000000
H	3.9810000000	-3.0710000000	-1.3290000000
C	1.7670000000	3.5780000000	3.6430000000
H	2.1500000000	3.1720000000	4.5740000000
C	5.2700000000	-3.3920000000	0.3700000000
H	6.1590000000	-3.5420000000	-0.2340000000
C	-5.4430000000	1.9580000000	0.3390000000
H	-6.0690000000	1.1800000000	0.7840000000
H	-6.1020000000	2.6750000000	-0.1620000000
H	-4.9290000000	2.4830000000	1.1490000000
C	-3.8460000000	-0.6420000000	1.0110000000
C	-0.3910000000	-4.0270000000	-1.8770000000
H	-1.0910000000	-3.1930000000	-1.9490000000
H	-0.9730000000	-4.9510000000	-1.9680000000
H	0.3000000000	-3.9830000000	-2.7240000000
C	-3.2530000000	2.6750000000	-1.6530000000
H	-2.5640000000	3.1160000000	-0.9290000000
H	-3.9700000000	3.4460000000	-1.9590000000

H	-2.6850000000	2.3930000000	-2.5430000000
C	1.4360000000	3.3720000000	1.2610000000
C	1.1260000000	4.8110000000	3.6330000000
Br	1.0080000000	5.3720000000	4.5540000000
C	2.9060000000	-2.9330000000	0.5170000000
C	1.9170000000	2.8580000000	2.4640000000
H	2.4070000000	1.8900000000	2.4590000000
C	4.3590000000	3.6650000000	0.2870000000
H	4.0110000000	4.5440000000	0.8350000000
H	5.2520000000	3.9510000000	-0.2790000000
H	4.6520000000	2.9050000000	1.0160000000
C	5.3500000000	-3.4660000000	1.7560000000
Br	6.2990000000	-3.6740000000	2.2380000000
C	-0.7050000000	-4.0660000000	1.1850000000
H	-0.1910000000	-4.0600000000	2.1510000000
H	-1.3190000000	-4.9730000000	1.1410000000
H	-1.3790000000	-3.2070000000	1.1450000000
C	-4.3790000000	-0.8390000000	3.3560000000
H	-4.3050000000	-0.4550000000	4.3680000000
C	1.5630000000	-5.6120000000	-0.1650000000
C	-5.1930000000	0.3840000000	-2.2700000000
C	2.6570000000	4.3590000000	-2.1570000000
H	3.5450000000	4.6350000000	-2.7350000000
H	2.2920000000	5.2610000000	-1.6560000000
H	1.8900000000	4.0290000000	-2.8650000000
H	2.3090000000	-5.6330000000	-0.9640000000
H	0.9120000000	-6.4840000000	-0.2880000000
H	2.0890000000	-5.7160000000	0.7870000000
H	-4.5210000000	-0.0540000000	-3.0150000000
H	-5.8570000000	1.0830000000	-2.7900000000
H	-5.8120000000	-0.4170000000	-1.8560000000

$(Me_3GeNPhBr)(Me_3Ge)_2P_7$

Charge = 0 Multiplicity = 1

Ge	4.5730020000	-0.9659720000	-1.2761190000
Ge	-1.2019750000	-3.0399210000	0.4711610000
Ge	0.6881610000	3.6881020000	0.0655410000
P	2.4826770000	-1.4033180000	-0.2687960000
P	-0.3762060000	0.1670760000	0.6738410000
P	0.8504120000	-0.2018970000	2.5148970000

P	2.8542560000	-0.9084720000	1.8629190000
P	1.1658000000	0.2298890000	-0.9251060000
P	2.2475450000	1.9105440000	-0.0300510000
P	2.4626620000	1.2663060000	2.1083310000
N	-1.4376660000	-1.1471210000	0.4600740000
C	-3.2497640000	-0.8472070000	-1.1684680000
H	-2.6064400000	-1.2651790000	-1.9355160000
C	1.8128170000	5.2790340000	-0.1293180000
H	2.5364810000	5.3458050000	0.6861410000
H	1.1868910000	6.1758070000	-0.1123640000
H	2.3560480000	5.2495950000	-1.0761970000
C	-0.3143560000	3.7762870000	1.7402730000
H	-1.0077850000	2.9364610000	1.8013110000
H	-0.8815870000	4.7117140000	1.7609300000
H	0.3528700000	3.7556350000	2.6046640000
C	-4.5399530000	-0.4438360000	-1.4901200000
H	-4.9165100000	-0.5588450000	-2.4995100000
C	-3.5755390000	-0.1265220000	1.0979310000
H	-3.1923710000	-0.0068030000	2.1059050000
C	-4.8614980000	0.2913790000	0.7878560000
H	-5.4926440000	0.7376140000	1.5470390000
C	5.4869020000	-2.6962790000	-1.2479390000
H	5.6240350000	-3.0474730000	-0.2228570000
H	6.4707550000	-2.5990380000	-1.7156540000
H	4.9097850000	-3.4417390000	-1.7989390000
C	4.2044880000	-0.4257960000	-3.1186300000
H	3.5724180000	-1.1613020000	-3.6205710000
H	5.1478750000	-0.3456880000	-3.6662760000
H	3.7024390000	0.5432500000	-3.1422710000
C	0.0752470000	-3.5520260000	1.8485340000
H	1.0998770000	-3.3354580000	1.5468090000
H	-0.0174690000	-4.6324330000	1.9955460000
H	-0.1377670000	-3.0564100000	2.7975620000
C	5.6299020000	0.3843730000	-0.3421790000
H	5.1059480000	1.3412260000	-0.3517150000
H	6.5918910000	0.4955650000	-0.8513350000
H	5.8156620000	0.0885790000	0.6926200000
C	-2.7547400000	-0.7033000000	0.1277310000
C	-2.9629490000	-3.7520220000	0.9193500000
H	-3.3113760000	-3.3378030000	1.8680370000

H	-2.8885350000	-4.8383800000	1.0212370000
H	-3.7012880000	-3.5242990000	0.1487990000
C	-0.5247900000	3.5295960000	-1.4594630000
H	0.0252350000	3.3725850000	-2.3894970000
H	-1.1029790000	4.4539680000	-1.5486660000
H	-1.2160950000	2.6981460000	-1.3100740000
C	-5.3373780000	0.1226030000	-0.5061470000
Br	-7.0983330000	0.6795420000	-0.9339830000
C	-0.6219350000	-3.6522020000	-1.2885100000
H	-1.3333440000	-3.3625330000	-2.0646130000
H	-0.5475760000	-4.7436210000	-1.2814610000
H	0.3609210000	-3.2401150000	-1.5247690000

6. Crystallography tables

Table S6. Crystallography data for benzyl azide inserted products **3** and **4**

Identification code	**3**	**4**
Empirical formula	$C_{30}H_{48}N_3P_7Si_3$	$C_{30}H_{48}Ge_3N_3P_7$
Formula weight / g mol^{-1}	751.77	885.27
Temperature / K	99.99(10)	99.99(10)
Crystal system	Triclinic	Triclinic
Space group	$P\bar{1}$	$P\bar{1}$
a/ Å	13.0673(3)	13.1543(3)
b/ Å	13.2072(3)	13.3096(4)
c/ Å	13.3756(2)	13.4195(2)
α /°	95.7235(16)	96.134(2)
β /°	101.4092(16)	100.642(2)
γ /°	116.974(2)	117.352(3)
Volume / Å^3	1968.62(8)	2000.84(10)
Z	2	2
ρ_{calc} / g cm^{-3}	1.268	1.469
μ / mm^{-1}	3.994	5.495
F(000)	792.0	900.0
Crystal size / mm^3	0.359 x 0.128 x 0.11	0.134 x 0.1 x 0.064
Radiation	Cu Kα (λ= 1.54184)	Cu Kα (λ= 1.54184)
2Θ range for data collection / °	7.694 to 151.446	6.87 to 151.658
Index ranges	$-16 \leq h \leq 16$, $-16 \leq k \leq 16$, $-14 \leq l \leq 16$	$-16 \leq h \leq 16$, $-16 \leq k \leq 16$, $-16 \leq l \leq 12$
Reflections collected	24699	26275
Independent reflections	7838 [R_{int} = 0.0158, R_{sigma} = 0.0161]	8049 [R_{int} = 0.0354, R_{sigma} = 0.0363]
Data/restraints/parameters	7838/0/397	8049/0/397
Goodness-of-fit on F^2	1.039	1.090
Final R indices [I>=2σ(I)]	R_1 = 0.0291, wR_2 = 0.0738	R_1 = 0.0380, wR_2 = 0.1000
Final R indices [all data]	R_1 = 0.0302, wR_2 = 0.0744	R_1 = 0.0428, wR_2 = 0.1027
Largest diff. peak/hole/ e Å^{-3}	0.80/–0.31	0.87/–0.72
CCDC	2351073	2351074

Table S7. Crystallography data for phenyl azide inserted products **5** and **6**.

Identification code	**5**	**6**
Empirical formula	$C_{27}H_{42}N_3P_7Si_3$	$C_{15}H_{32}Ge_3NP_7$
Formula weight / g mol^{-1}	709.80	660.97
Temperature / K	100.00(10)	100.0(3)
Crystal system	Trigonal	Triclinic
Space group	*R*3*c*	$P\bar{1}$
a/ Å	22.0812(2)	9.8517(2)
b/ Å	22.0812(2)	11.4406(3)
c/ Å	66.4303(5)	13.6986(3)
α /°	90	99.425(2)
β /°	90	108.719(2)
γ /°	120	103.252(2)
Volume / Å^3	28050.6(6)	1375.38(6)
Z	30	2
ρ_{calc} / g cm^{-3}	1.261	1.596
μ / mm^{-1}	4.177	7.747
F(000)	11162.0	660.0
Crystal size / mm^3	0.194 x 0.157 x 0.137	0.153 x 0.082 x 0.057
Radiation	Cu Kα (λ= 1.54184)	Cu Kα (λ= 1.54184)
2Θ range for data collection / °	5.332 to 153.088	7.052 to 151.55
Index ranges	$-19 \le h \le 27, -27 \le k \le 27, -78 \le l \le 81$	$-12 \le h \le 12, -14 \le k \le 14, -16 \le l \le 14$
Reflections collected	94219	15616
Independent reflections	12613 [R_{int} = 0.0398, R_{sigma} = 0.0283]	5494 [R_{int} = 0.0242, R_{sigma} = 0.0271]
Data/restraints/parameters	12613/1445/825	5494/0/244
Goodness-of-fit on F^2	1.031	1.029
Final R indices [I>=2σ (I)]	R_1 = 0.0460, wR_2 = 0.1245	R_1 = 0.0216, wR_2 = 0.0578
Final R indices [all data]	R_1 = 0.0469, wR_2 = 0.1256	R_1 = 0.0240, wR_2 = 0.0588
Largest diff. peak/hole/ e Å^{-3}	0.83/–0.83	0.51/–0.45
CCDC	2351075	2351076

Table S8. Crystallography data for 4-bromophenyl azide inserted products **8** and **9**.

Identification code	**8**	**9**
Empirical formula	$C_{27}H_{39}Br_3N_3P_7Si_3$	$C_{30}H_{62}Br_2Ge_6N_2P_{14}$
Formula weight / g mol^{-1}	949.40	1479.75
Temperature / K	99.97(18)	100.0(2)
Crystal system	Trigonal	Monoclinic
Space group	*R*3	$P2_1/c$
a/ Å	23.3336(3)	19.55440(10)
b/ Å	23.3336(3)	11.53790(10)
c/ Å	13.0832(2)	26.14240(10)
α /°	90	90
β /°	90	98.93
γ /°	120	90
Volume / Å^3	6168.90(18)	5826.68(6)
Z	6	4
ρ_{calc} / g cm^{-3}	1.592	1.687
μ / mm^{-1}	7.240	8.934
F(000)	2844.0	2912.0
Crystal size / mm^3	0.088 x 0.123 x 0.149	0.091 x 0.122 x 0.170
Radiation	Cu Kα (λ= 1.54184)	Cu Kα (λ= 1.54184)
2Θ range for data collection / °	8.05 to 152.73	4.574 to 152.426
Index ranges	$-26 \le h \le 29$, $-29 \le k \le 27$, $-16 \le l \le 15$	$-24 \le h \le 24$, $-14 \le k \le 14$, $-32 \le l \le 32$
Reflections collected	21122	68522
Independent reflections	5196 [R_{int} = 0.0336, R_{sigma} = 0.0231]	12079 [R_{int} = 0.0406, R_{sigma} = 0.0268]
Data/restraints/parameters	5196/1/265	12079/0/505
Goodness-of-fit on F^2	1.064	1.084
Final R indices [I>=2σ (I)]	R_1 = 0.0290, wR_2 = 0.0733	R_1 = 0.0226, wR_2 = 0.0535
Final R indices [all data]	R_1 = 0.0292, wR_2 = 0.0734	R_1 = 0.0250, wR_2 = 0.0545
Largest diff. peak/hole/ e Å^{-3}	0.66/–0.71	0.45/–0.545
CCDC	2351077	2351078

7. References

(1) Fritz, G.; Hoppe, K. D.; Hönle, W.; Weber, D.; Mujica, C.; Manriquez, V.; Schnering, H. G. v. Substituted Heptaphosphanortricyclenes: Derivatives and Homologues of $P_7(SiMe_3)_3$. *J. Organomet. Chem.* **1983**, *249* (1), 63–80.
(2) Kuveke, R. E. H.; Barwise, L.; van Ingen, Y.; Vashisth, K.; Roberts, N.; Chitnis, S. S.; Dutton, J. L.; Martin, C. D.; Melen, R. L. An International Study Evaluating Elemental Analysis. *ACS Cent. Sci.* **2022**, *8* (7), 855–863.
(3) Sheldrick, G. M. *Acta. Cryst.* **2015**, *A71*, 3–8. Dolomanov, O. V.; Bourhis, L. J.; Gildea, R. J.; Howard, J. A. K.; Puschmann, H. *J. Appl. Cryst.* **2009**, *42*, 339–341.
(4) NBO Version 3.1, E. D. G., A. E. Reed, J. E. Carpenter, and F. Weinhold.
(5) a) Hohenberg, P.; Kohn, W. Inhomogeneous Electron Gas. *Phys. Rev.* **1964**, *136* (3B), B864–B871. b) Kohn, W.; Sham, L. J. Self-Consistent Equations Including Exchange and Correlation Effects. *Phys. Rev.* **1965**, *140* (4A), A1133–A1138. c) Peng, C.; Ayala, P. Y.; Schlegel, H. B.; Frisch, M. J. Using Redundant Internal Coordinates to Optimize Equilibrium Geometries and Transition States. *J. Comp. Chem.* **1996**, *17* (1), 49–56. d) Gaussian 09, R. A., M. J. Frisch, G. W. Trucks, H. B. Schlegel, G. E. Scuseria, M. A. Robb, J. R. Cheeseman, G. Scalmani, V. Barone, G. A. Petersson, H. Nakatsuji, X. Li, M. Caricato, A. Marenich, J. Bloino, B. G. Janesko, R. Gomperts, B. Mennucci, H. P. Hratchian, J. V. Ortiz, A. F. Izmaylov, J. L. Sonnenberg, D. Williams-Young, F. Ding, F. Lipparini, F. Egidi, J. Goings, B. Peng, A. Petrone, T. Henderson, D. Ranasinghe, V. G. Zakrzewski, J. Gao, N. Rega, G. Zheng, W. Liang, M. Hada, M. Ehara, K. Toyota, R. Fukuda, J. Hasegawa, M. Ishida, T. Nakajima, Y. Honda, O. Kitao, H. Nakai, T. Vreven, K. Throssell, J. A. Montgomery, Jr., J. E. Peralta, F. Ogliaro, M. Bearpark, J. J. Heyd, E. Brothers, K. N. Kudin, V. N. Staroverov, T. Keith, R. Kobayashi, J. Normand, K. Raghavachari, A. Rendell, J. C. Burant, S. S. Iyengar, J. Tomasi, M. Cossi, J. M. Millam, M. Klene, C. Adamo, R. Cammi, J. W. Ochterski, R. L. Martin, K. Morokuma, O. Farkas, J. B. Foresman, and D. J. Fox, Gaussian, Inc., Wallingford CT, 2016.
(6) a) Perdew, J. P.; Burke, K.; Ernzerhof, M. Generalized Gradient Approximation Made Simple. *Phys. Rev. Lett.* **1996**, *77* (18), 3865–3868. b) Perdew, J. P.; Ernzerhof, M.; Burke, K. Rationale for Mixing Exact Exchange with Density Functional Approximations. *J. Chem. Phys.* **1996**, *105* (22), 9982–9985. c) Krishnan, R.; Binkley, J. S.; Seeger, R.; Pople, J. A. Self-Consistent Molecular Orbital Methods. XX. A Basis Set For Correlated Wave Functions. *J. Chem. Phys.* **1980**, *72* (1), 650–654.
(7) Lu, T.; Chen, F. Multiwfn: A Multifunctional Wavefunction Analyzer. *J. Comp. Chem.* **2012**, *33* (5), 580–592.
